# Major, trace and rare earth elements of apatite and zircon U-Pb ages of ore-associated and barren granitoids from the Edong ore district, South China

**DOI:** 10.1016/j.dib.2018.08.154

**Published:** 2018-09-01

**Authors:** Deng-Fei Duan, Shao-Yong Jiang

**Affiliations:** State Key Laboratory of Geological Processes and Mineral Resources, Collaborative Innovation Centre for Exploration of Strategic Mineral Resources, Faculty of Earth Resources, China University of Geosciences, Wuhan 430074, PR China

**Keywords:** Major elements, Volatile elements, Trace and rare earth elements, U-Pb date, Apatite, Zircon, Granitoids

## Abstract

The data in this article contains major, trace and rare earth element concentrations of apatite, and statistic analysis of apatite morphology in the granitoid rocks from the Edong ore district, South China, together with zircon U-Pb dating results for these rocks. Both the ore-associated and barren granitoids yield similar ages, including quartz diorite in the Fuzishan Cu-Au skarn deposit (138.6 ± 2.9 Ma), quartz monzodiorite in the Niutoushan Cu skarn deposit (137.8 ± 1.8 Ma), quartz diorite in the Ouyangshan Cu skarn deposit (138.4 ± 1.2 Ma), Liujiawan quartz monzodiorite (135.0 ± 2.4 Ma), and Bengqiaodi quartz monzodiorite porphyry (138.7 ± 1.1 Ma). Apatite occurs in all the minerals for each rock sample, and a detailed petrographic analysis shows the majority of them are early formed mineral phase. The F, Cl, SO_3_ contents in apatite are different, those in ore-associated rocks show higher values than barren ones. Li, Ni, Co, V contents are also higher in apatites from the ore-associated rocks than in barren rocks. Apatite (La/Sm)_N_ and (Yb/Sm)_N_ ratio show a positive correlation for ore-associated rocks but a negative correlation for barren rocks.

**Specifications table**

**Table:** 

Subject area	*Mineralogy, geochemistry, ore deposit*
More specific subject area	*Concentration of major and trace elements in apatite and host granitic rocks and zircon U-Pb dating*
Type of data	*Table and figure*
How data was acquired	*Electron Microscope (*JEOL JXA-8230, Japan*), Laser Abalation System (*Resolution-M50 193 nm UV ArF Excimer, Australian*),* Inductively Coupled Plasma Mass Spectrometer (ThermoiCAP-Q ICP-MS, Germany)
Data format	*Raw and analyzed*
Experimental factors	*Apatite and zircon crystals were separated from crushed fresh granitoid samples. Cathodoluminescence (CL) images were taken before the LA-ICP-MS analyses.*
Experimental features	*First, the samples were subjected to magnetic separation and flotation. Then, representative sets of clean apatite and zircon grains were selected by hand under the microscope, and arranged on the epoxy resin sample disks. Finally, the prepared and polished sample disks were analyzed.*
Data source location	*Wuhan, China University of Geosciences, China*
Data accessibility	*Data is with this article.*
Related research article	*The data article is submitted as a companion paper to a research article, Duan DF and Jiang SY, in press, Using apatite to discriminate synchronous ore-associated and barren granitic rocks: A case study from the Edongmetallogenic district, South China. Lithos*[1]

**Value of the data**

• The dataset includes a wide range of major and trace elements in apatite both from ore-associated and barren granitoids.
• The different concentrations of the elements in apatite from the ore-associated and barren granitoids can serve as good indicators for mineral exploration.
• Correlations of a number of elements in apatites and their whole rocks indicate a magma chemistry control for some components.
• The data could be used to assess the ore potential of an unknown granitic pluton.
• The dataset is very useful to prospecting geochemists as an indicator for taking further necessary steps to explore skarn and porphyry type deposits.

## 1 Data

We sampled granitoid samples from three Cu or Cu-Au skarn ore deposits and two barren plutons from different depths of drill holes or underground tunnels in the Edong ore district, South China. The ore-associated rocks are collected from Fuzishan Cu-Au skarn deposit, Niutoushan Cu skarn deposit, and Ouyangshan Cu skarn deposit. The barren rocks are from Liujiawan quartz monzodiorite and Bengqiaodi quartz monzodiorite porphyry.

**Fig. 1:**
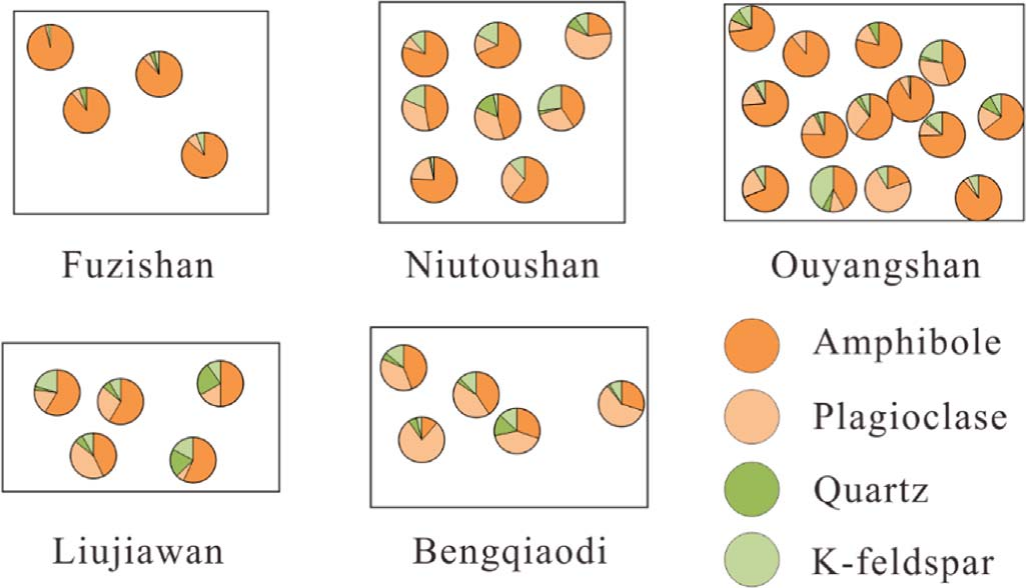
Schematic maps of the apatite abundance statistic.

A detailed petrographic analysis is carried out for all the rock types. The corresponding samples for the thin sections examination for the apatite abundance statistical study were also used to separate the apatite and zircon, and the results are shown in Fig. 1. In this figure, the rectangle is the boundary of each sample in thin section. Each circle represents one statistical area. The diameter of the circle is ~5 mm. Apatite has been observed in all the minerals for each sample. But amphibole, plagioclase, quartz and K-feldspar composes about 95% of the bulk rock. So we only count the apatite in these four kinds of minerals. The apatite can be roughly divided into two kinds—the early phase (in amphibole and plagioclase) and late phase (in quartz and K-feldspar).

There are also a few interstitial apatites distributed within the interval of the plagioclase and amphibole. These apatites crystallized later than those in amphibole and plagioclase. We ascribe this kind of apatites to quartz or K-feldspar randomly whenever we observe it. This does not affect the final result considering the apatite classification.

Although Liujiawan and Bengqiaodi have 3% and 5% biotite respectively, we did not count the apatites in the biotite. One important reason is that biotite does not distribute evenly in the thin section like amphibole, plagioclase, quartz and K-feldspar. But excluding this part of data has little effect on the final statistic results due to the low apatite abundance in biotite.

In summary, the statistic results can represent the relative abundance of apatite in different minerals. Results show that 95.6%, 84.4%, 90.4%, 76.1%, 84.3% of apatites crystallized early in Fuzishan, Niutoushan, Ouyangshan, Liujiawan and Bengqiaodi respectively. Therefore, we regarded that the majority of apatite is an early phase in all the studied samples.

Zircon U-Pb dating show all the studied rocks have similar ages (Table 1). The quartz diorite in the Fuzishan Cu-Au skarn deposit yields an age of 138.6 ± 2.9 Ma, the quartz monzodiorite in the Niutoushan Cu skarn deposit yields an age of 137.8 ± 1.8 Ma, and the quartz diorite in the Ouyangshan Cu skarn deposit gives an age of 138.4 ± 1.2 Ma. Similarly, the Liujiawan quartz monzodiorite and Bengqiaodi quartz monzodiorite porphyry show ages of 135.0±2.4 Ma and 138.7 ± 1.1 Ma respectively.

Major element concentrations including SiO_2_, FeO, MgO, MnO, CaO, Na_2_O, P_2_O_5_, SO_3_, F and Cl of apatite were determined by electron microprobe (Table 2). Apatite contains mainly CaO (53.06–54.89 wt%), P_2_O_5_ (40.29–42.50 wt%) and F((1.49–4.23 wt%). The concentrations of FeO, MgO, BaO, K_2_O and Al_2_O_3_ are all low and mostly below the detection limit. Apatite from the ore-associated rocks has higher Cl (0.19–0.57 wt%, average 0.35 wt%) and lower F (1.49–3.18 wt%, average 2.34 wt%) than barren ones (0.09–0.31 wt%, average 0.16 wt% Cl, 1.96–4.23 wt%, average 2.87 wt% F). The content of SO_3_ are higher in apatite from the ore-associated rocks (0.08–0.71 wt%, average 0.32 wt%) than the barren ones(0.06–0.28 wt%, average 0.16 wt%) although with some overlap.

Trace element concentrations including Li, B, Sc, V, Cr, Mn, Co, Ni, Ga, Ge, Rb, Sr, Y, Zr, Ba, REE, Hf, Ta, W, Pb, Th and U in apatite are analyzed by LA-ICP-MS (Table 3). Apatite from the ore-associated rocks shows higher contents of Li (0.49–7.99 ppm), Ni (1.3–2.1 ppm), Co (0.16–0.55 ppm) and V (11–47 ppm) and lower Sc (0.01–0.38 ppm) than the barren rocks (0.15–0.90 ppm Li, 1.1–1.7 ppm Ni, 0.17–0.35 ppm Co, 10–16 ppm V, 0.34–1.03 ppm Sc).

Apatites from both the ore-associated and barren rocks show similar REE patterns and are enriched in LREE compared with HREE. Apatites from ore-associated rocks show a relatively higher and larger variation of (La/Yb)_N_ and Eu/Eu^*^ than barren rocks. Apatite (La/Sm)_N_ and (Yb/Sm)_N_ ratio show a positive correlation for ore-associated rocks but a negative correlation for barren rocks.

Major and trace element concentrations of the studied granitoids including Fuzishan, Niutoushan, Quyangshan, Bengqiaodi and Liujiawan are listed in Table 4.

**Fig. 2:**
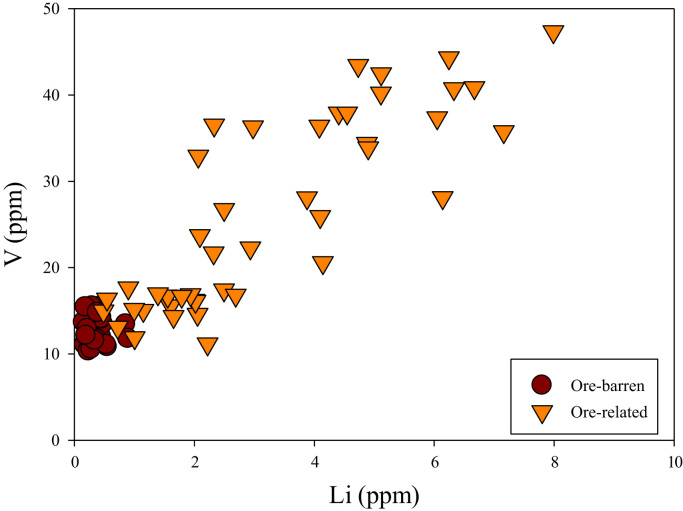
Li versus V diagram.

**Fig. 3:**
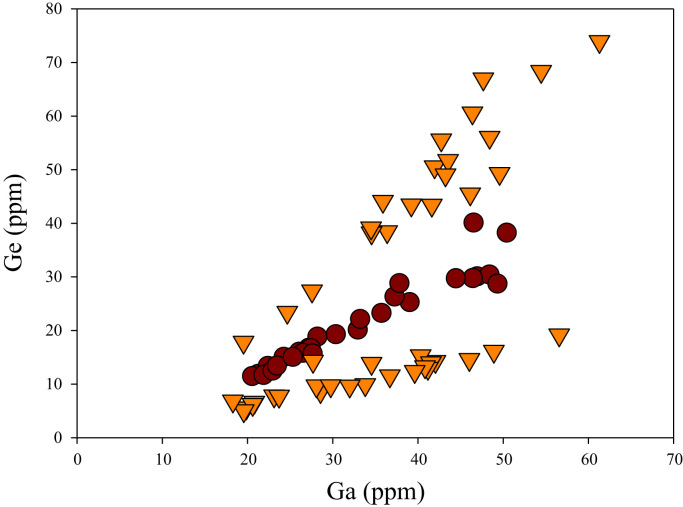
Ga versus Ge diagram.

**Fig. 4:**
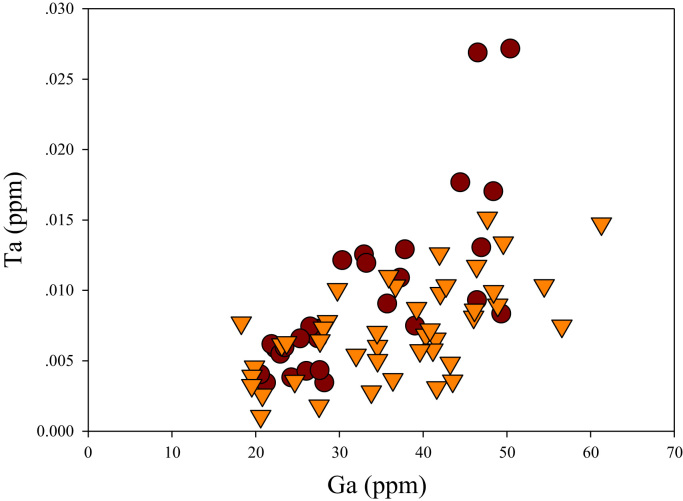
Ga versus Ta diagram.

**Fig. 5:**
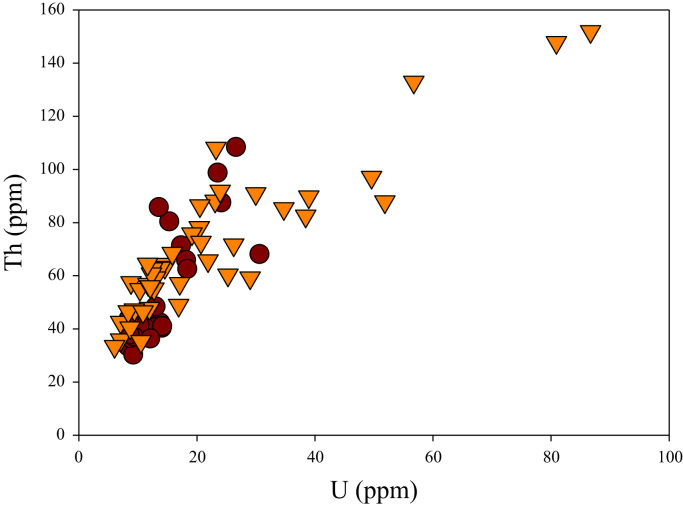
U versus Th diagram.

**Fig. 6:**
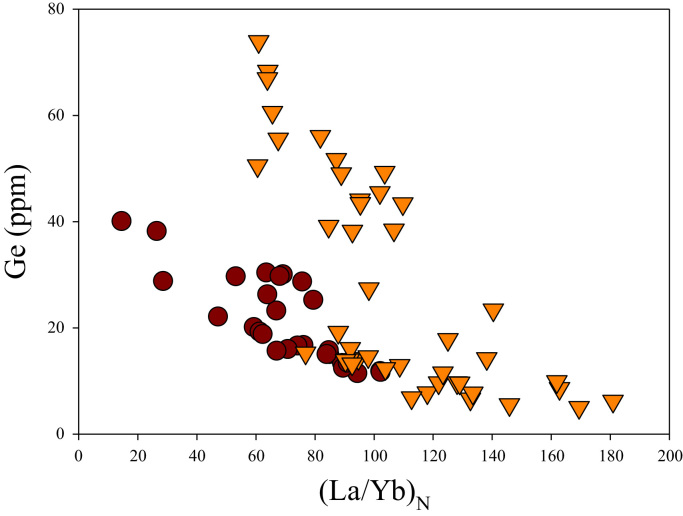
(La/Yb)_N_ versus Ge diagram.

**Fig. 7:**
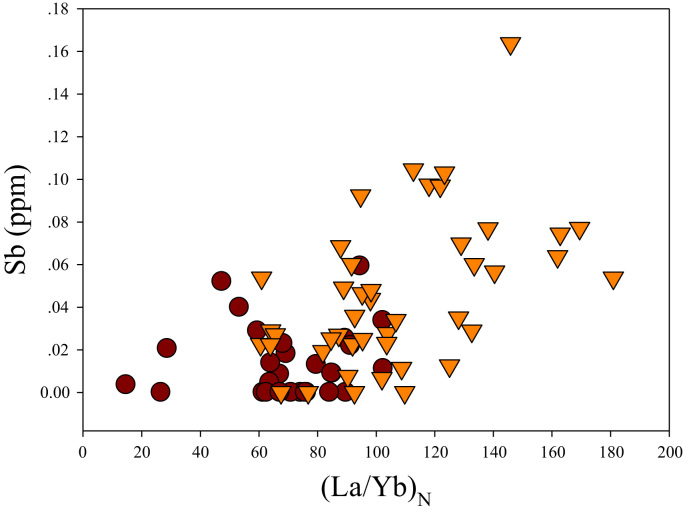
(La/Yb)_N_ versus Sb diagram.

**Fig. 8:**
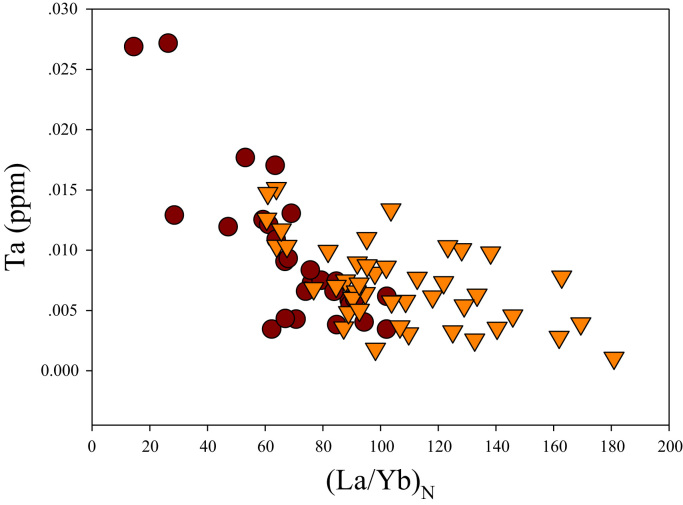
(La/Yb)_N_ versus Ta diagram.

**Fig. 9:**
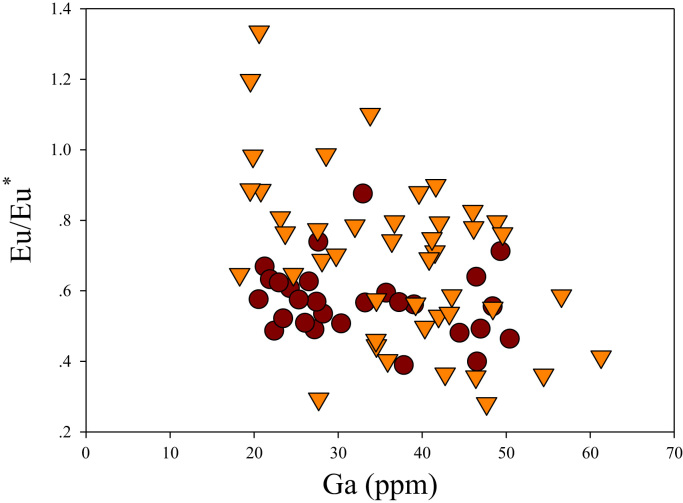
Ga versus Eu/Eu^*^ diagram.

**Fig. 10:**
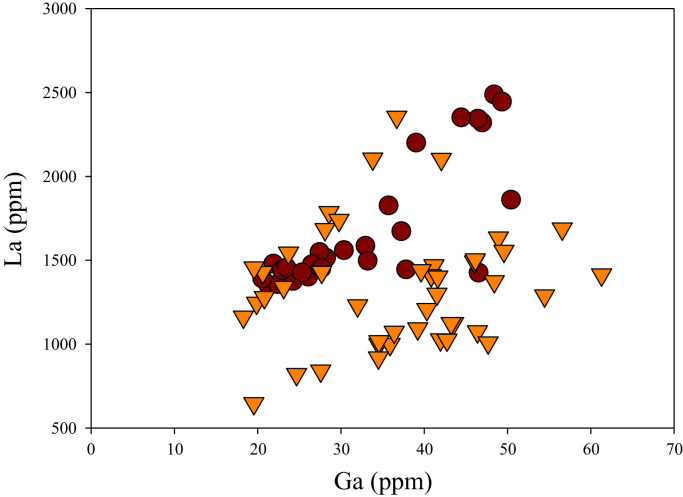
Ga versus La diagram.

**Fig. 11:**
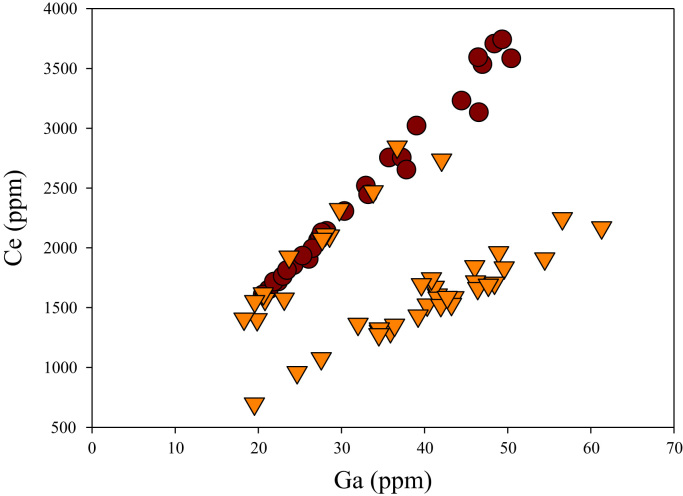
Ga versus Ce diagram.

**Fig. 12:**
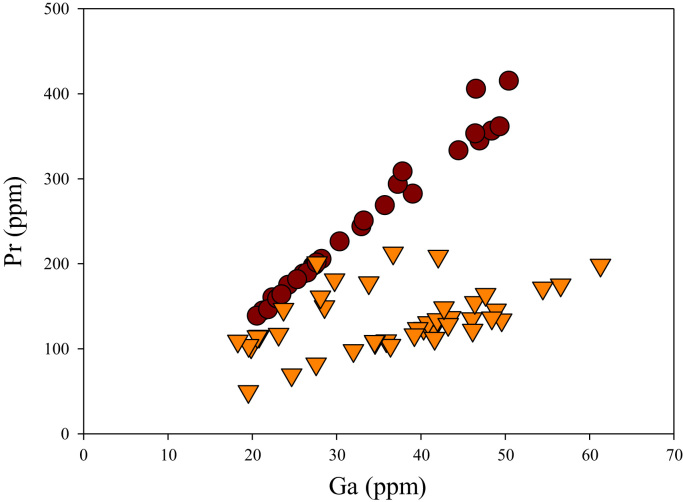
Ga versus Pr diagram.

**Fig. 13:**
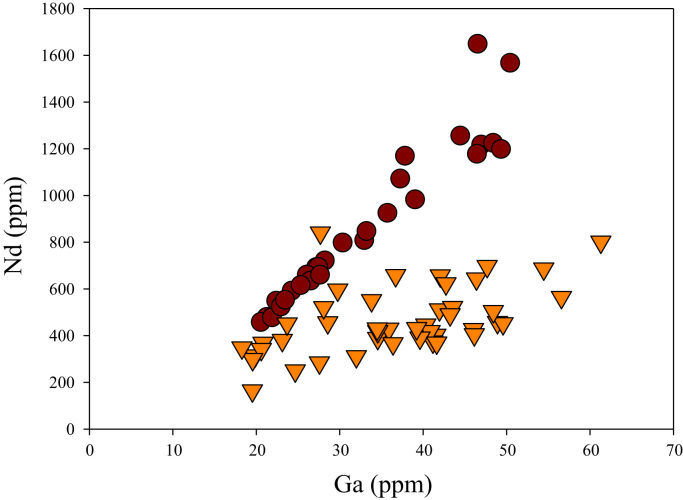
Ga versus Nd diagram.

**Fig. 14:**
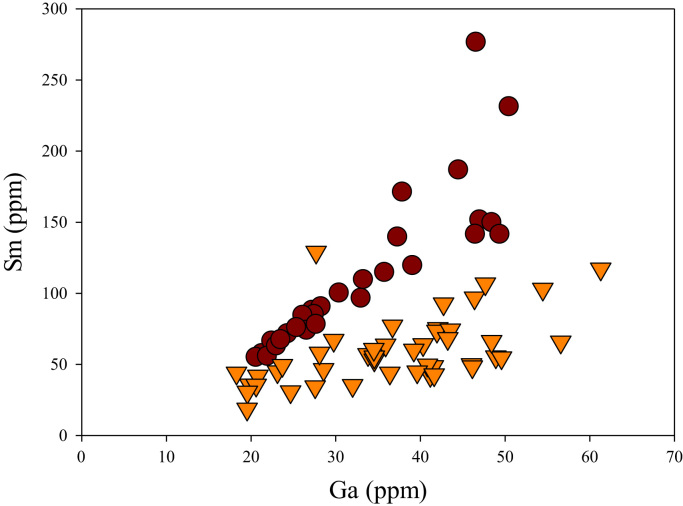
Ga versus Sm diagram.

**Fig. 15:**
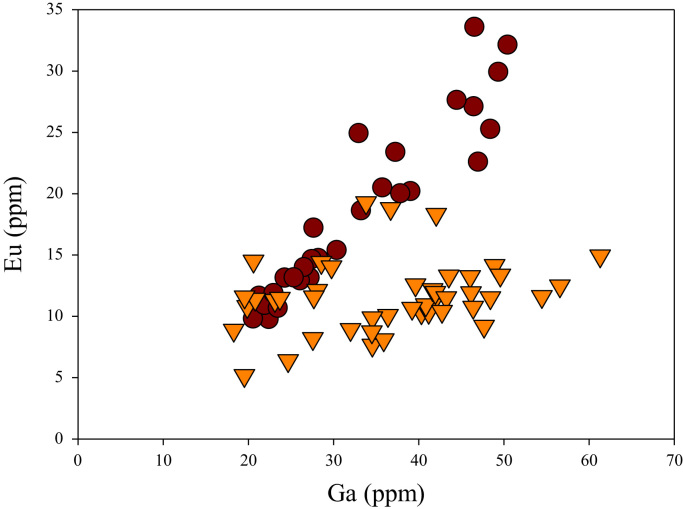
Ga versus Eu diagram.

**Fig. 16:**
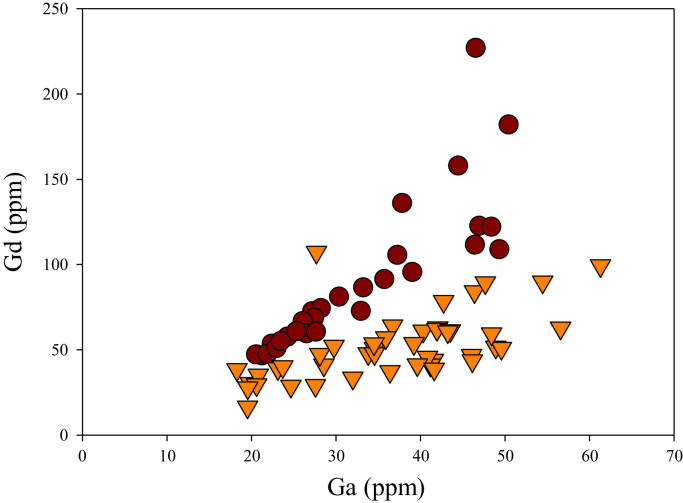
Ga versus Gd diagram.

**Fig. 17:**
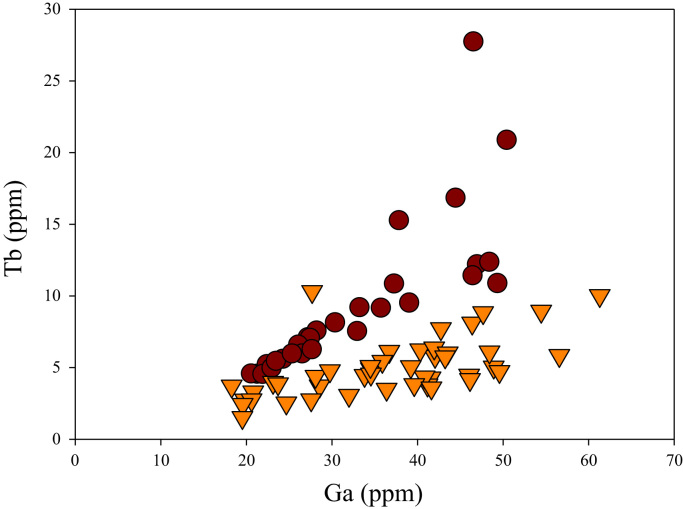
Ga versus Tb diagram.

**Fig. 18:**
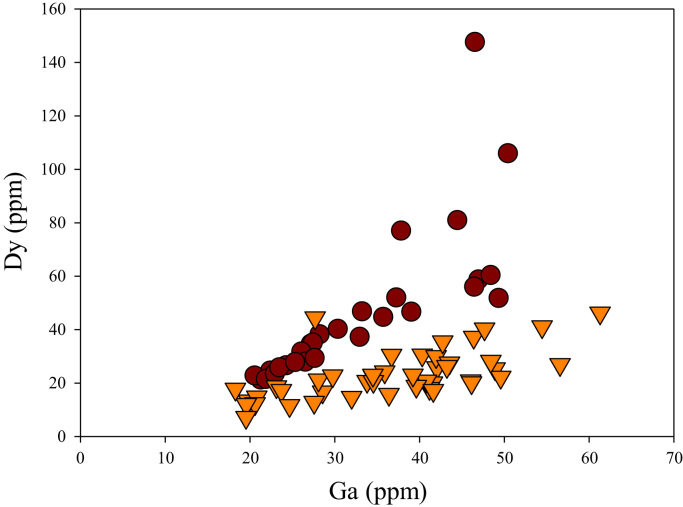
Ga versus Dy diagram.

**Fig. 19:**
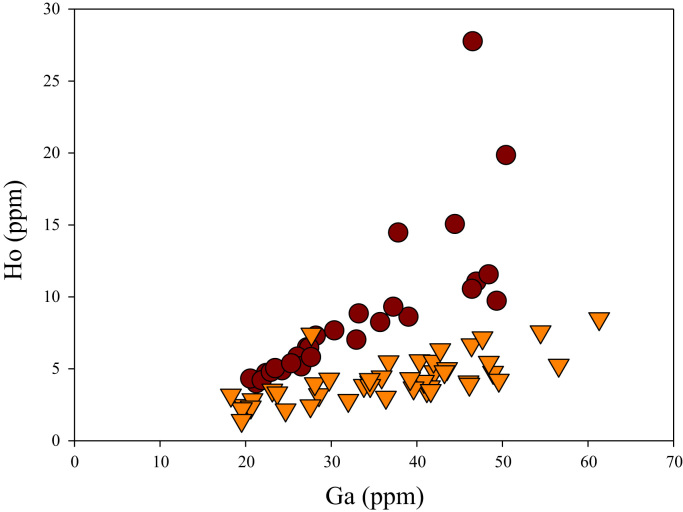
Ga versus Ho diagram.

**Fig. 20:**
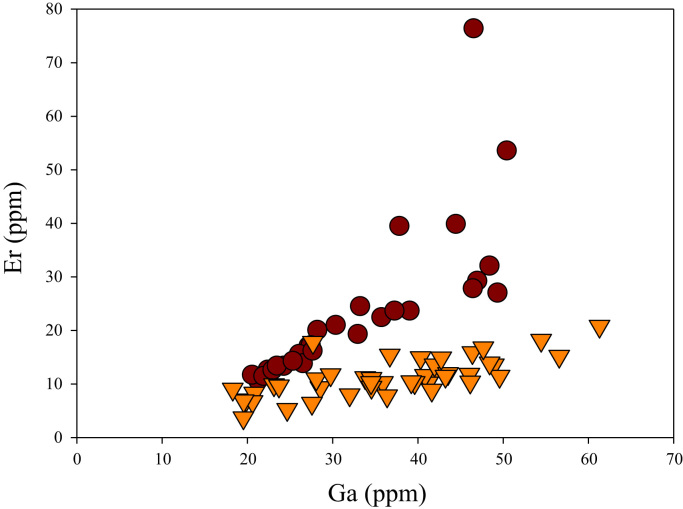
Ga versus Er diagram.

**Fig. 21:**
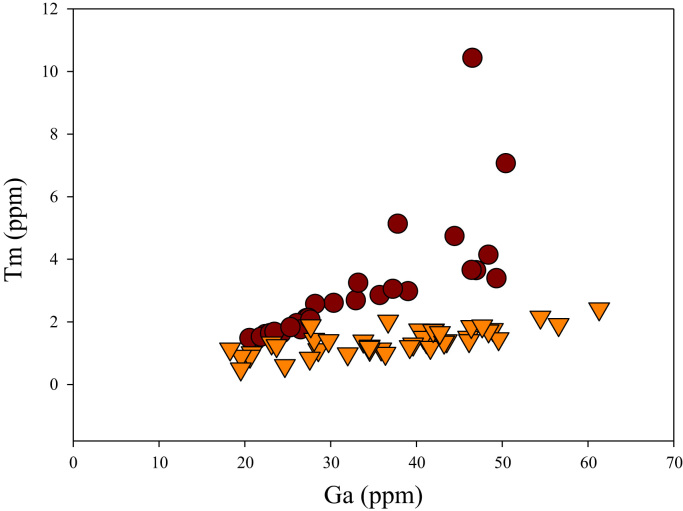
Ga versus Tm diagram.

**Fig. 22:**
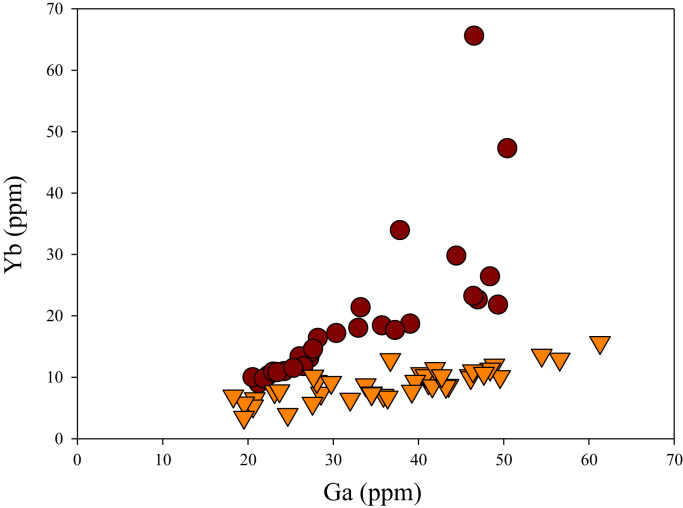
Ga versus Yb diagram.

**Fig. 23:**
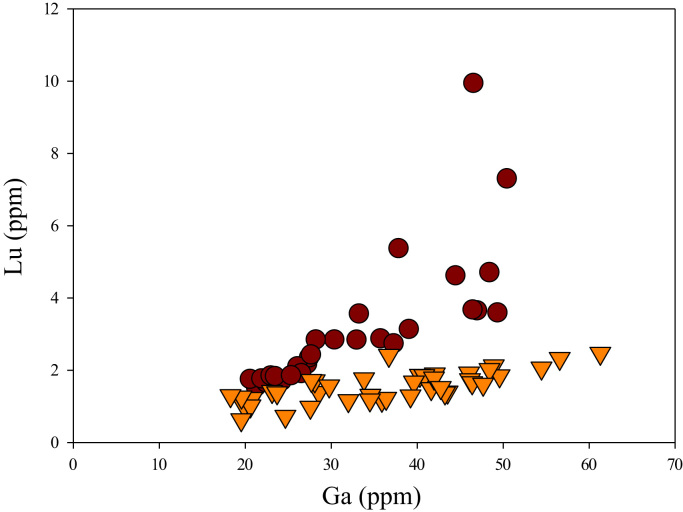
Ga versus Lu diagram.

**Fig. 24:**
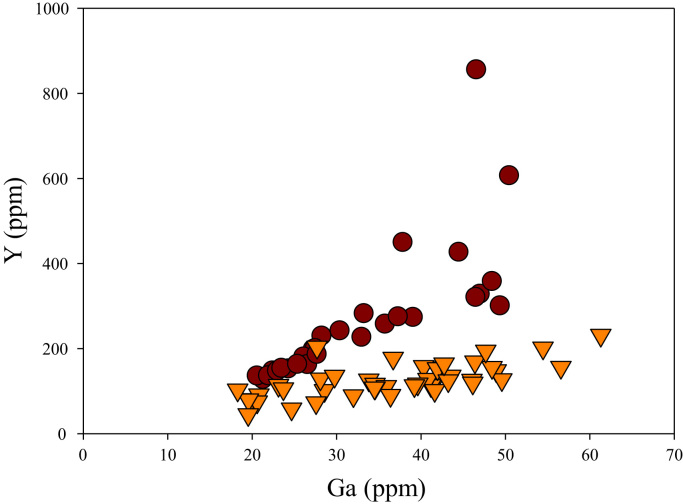
Ga versus Y diagram.

A correlation between different element pairs in apatites from ore-associated and barren rocks is shown in Fig. 2, Fig. 3, Fig. 4, Fig. 5, Fig. 6, Fig. 7, Fig. 8, Fig. 9, Fig. 10, Fig. 11, Fig. 12, Fig. 13, Fig. 14, Fig. 15, Fig. 16, Fig. 17, Fig. 18, Fig. 19, Fig. 20, Fig. 21, Fig. 22, Fig. 23, Fig. 24. Fig. 2 shows V vs Li concentrations in apatite. The ore-associated samples show higher V and Li concentrations. Fig. 3 shows Ga vs Ge concentrations in apatite. Both have similar range for Ga concentrations, but the ore-associated samples have higher and wider variations of Ge contents. Fig. 4 shows Ga vs Ta concentrations in apatite. Both dataset are similar, although a few data for barren samples show slightly higher Ta concentrations. Fig. 5 shows U vs Th concentrations in apatite. It seems parts of the ore-associated samples show higher U and Th contents. Fig. 6 shows (La/Yb)_N_ ratios vs Ge contents in apatite. The ore-associated samples show higher (La/Yb)_N_ ratios and larger Ge variation. Fig. 7 shows (La/Yb)_N_ ratios vs Sb contents in apatite. The ore-associated samples show higher (La/Yb)_N_ ratios and larger Sb variation. Fig. 8 shows (La/Yb)_N_ ratios vs Ta contents in apatite. The ore-associated samples show higher (La/Yb)_N_ ratios but lower and limited Ta variation. Fig. 9 shows Eu/Eu* ratios vs Ga contents in apatite. The ore-associated samples show higher Eu/Eu* ratios but similar Ga variation.

Fig. 10 shows Ga vs La contents in apatite. Both are similar although parts of the data from ore-associated samples have lower La concentrations. Fig. 11 shows Ga vs Ce contents in apatite. Good linear correlations are observed for both sample sets, but parts of the ore-associated samples show lower Ce concentrations at the same Ga contents. Similar trend is also observed for Ga vs Pr, Ga vs Nd, Ga vs Sm, Ga vs Eu, Ga vs Gd, Ga vs Tb, Ga vs Dy, Ga vs Ho, Ga vs Er, Ga vs Tm, Ga vs Yb, Ga vs Lu, and Ga vs Y, as shown in Fig. 12, Fig. 13, Fig. 14, Fig. 15, Fig. 16, Fig. 17, Fig. 18, Fig. 19, Fig. 20, Fig. 21, Fig. 22, Fig. 23, Fig. 24.

**Fig. 25:**
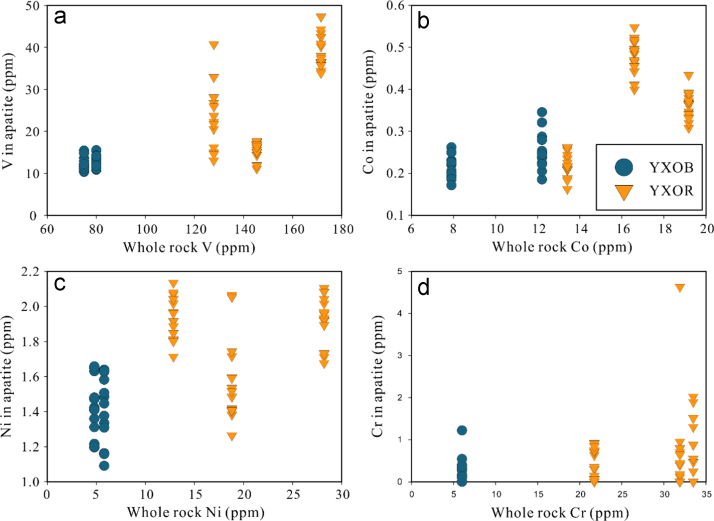
Relationships between V, Co, Ni and Cr in apatite and whole rock composition (a-d).

In Fig. 25, a relationship between V, Co, Ni and Cr in apatite and whole rock compositions is displayed. Compatible elements such as Co, Ni and V in ore-associated rocks are higher than in barren granitoids, but there is little correlation between V, Co and Ni contents in apatite and the composition of bulk rock as sampled for this study (Fig. 25). Sc in the apatite even has an opposite variation trend (Fig. 25).

## 2 Experimental design, materials, and methods

Both apatite and zircon crystals were separated from the studied granitoid samples in the Edong ore district, South China. The crushing rock samples were subjected to magnetic separation and flotation to separate zircon and apatite mineral grains, and epoxy resin sample disks were prepared for further use for electron microprobe and LA-ICP-MS analyses.

### 2.1 U-Pb zircon dating

Instrument used is a Resolution-M50 193 nm UV ArF Excimer laser coupled to a Thermo iCAP-Q Inductively Coupled Plasma Mass Spectrometer (LA-ICP-MS) at State Key Laboratory of Geological Processes and Mineral Resources, China University of Geosciences, Wuhan. The standards used include Zircon 91500 and NIST SRM 612. We applied a ‘standard-sample bracketing’ (SSB) technique for zircon dating, and the standard was analyzed twice every 5–8 spot analyses. Both standards and samples were ablated for 30 s with a 33 μm-diameter beam at a pulse rate of 10 Hz. The ^206^Pb/^238^U age of Zircon 91500 is 1065 ± 0.6 Ma [6]. The standard NIST SRM 612 was used to correct the instrumental drift. Off-line selection, integration of background and analytic signals, time-drift correction and quantitative calibration for trace element analyses and U-Pb dating were performed using ICPMSDataCal software [3], [4]. The concordia diagram and weighted mean U-Pb ages were plotted and calculated using the ISOPLOT/EX 3 software package [5].

### 2.2 Electron Microprobe Analysis (EMPA) for major elements of apatite

Instrument used is a JEOL JXA-8230 electron microprobe equipped with four wave spectrometers at the Testing Center of the China Metallurgical Geological Bureau, Shandong. During the analysis, 15 kV acceleration voltage, acceleration 20 nA current and 5 μm spot beam were applied. The measurement time of the characteristic peaks for Ca and P was 10 s, and 20 s for Na, Mg, Si, Fe, Mn, Sr, F and Cl. The measurement time of the upper and lower background was half of the measurement time of the peaks. We select the apatite for analysis that was polished in direction perpendicular to apatite׳s c axis to avoid the possible large error in compositional variation (e.g., [2]). All analytical data were corrected by the ZAF (atomic number, absorption, fluorescence) method. The standards used were apatite (Ca, P), jadeite (Na, Si), diopside (Mg), olivine (Fe), rhodonite (Mn), celestite (Sr), phlogopite (F) and tugtupite (Cl). Precision for EMPA analysis was calculated from counting statistics, and was generally better than ± 1% for measurements > 10 wt%, and better than ± 5% for contents > 0.5 wt%.

### 2.3 LA-ICP-MS analysis for trace and rare earth elements of apatite

The trace and rare earth element concentrations of apatite were measured in the same lab as U-Pb zircon dating but using a 193 nm Excimer ArF laser ablation system. The standards used include NIST SRM 612 (to correct for signal drift) and the international basalt glass standards NIST BCR-2G, BHVO-2G and BIR-1G (as external standards). Ca content in apatite measured by electron microprobe was used as the internal standard to calibrate the trace elements. The raw data were processed off-line by ICPMSDataCal software, version 9.9 [3]. The relative error is less than 10% for Li, Sc, V, Cr, Co, Ni, Cu, Zn, Ga, Ge, Sr, Y, Zr, Nb, Sn, Ba, REE, Hf in BIR-1G, BHVO-2G and BCR-2G but is 22%, 16%, 20% and 19% for Rb, Ta, Th and U in BIR-1G respectively and <5% in BHVO-2G and BCR-2G.
