# Supplementary material for: Major, trace and rare earth elements of apatite and zircon U-Pb ages of ore-associated and barren granitoids from the Edong ore district, South China
Source: Data Brief. 2018 Sep 1;20:1587–601. doi: 10.1016/j.dib.2018.08.154 (PMC6153191; doi:10.1016/j.dib.2018.08.154)
Supplement: Supplementary file 2 — Supplementary material [file mmc2.docx]

**Table 1**

LA-ICP-MS zircon U-Pb data of Fuzishan, Niutoushan, Ouyangshan, Liujiawan and Bengqiaodi granites from Edong district, south China

| Spot | Isotope ratios | | | | | | Age (Ma) | | | |
| --- | --- | --- | --- | --- | --- | --- | --- | --- | --- | --- |
|  | ^207^Pb/^206^Pb | ±1σ | ^207^Pb/^235^U | ±1σ | ^206^Pb/^238^U | ±1σ | ^207^Pb/^235^U | ±1σ | ^206^Pb/^238^U | ±1σ |
| Fuzishan Quartz diorite, weighted mean ^206^Pb/^238^U age = 138.6 ± 2.9 Ma | | | | | | | | | | |
| FZ-12-1 | 0.0471 | 0.0032 | 0.1457 | 0.0100 | 0.0226 | 0.0004 | 138.1 | 8.9 | 144.0 | 2.3 |
| FZ-12-2 | 0.0463 | 0.0031 | 0.1358 | 0.0085 | 0.0215 | 0.0004 | 129.3 | 7.6 | 137.3 | 2.2 |
| FZ-12-3 | 0.0493 | 0.0018 | 0.1436 | 0.0052 | 0.0213 | 0.0002 | 136.2 | 4.6 | 135.7 | 1.4 |
| FZ-12-4 | 0.0460 | 0.0014 | 0.1373 | 0.0043 | 0.0218 | 0.0002 | 130.7 | 3.8 | 139.1 | 1.5 |
| FZ-12-5 | 0.0480 | 0.0026 | 0.1444 | 0.0080 | 0.0218 | 0.0003 | 136.9 | 7.1 | 139.1 | 2.0 |
| FZ-12-6 | 0.0521 | 0.0020 | 0.1611 | 0.0075 | 0.0221 | 0.0004 | 151.6 | 6.6 | 141.2 | 2.4 |
| FZ-12-7 | 0.0472 | 0.0017 | 0.1383 | 0.0052 | 0.0212 | 0.0002 | 131.5 | 4.7 | 135.4 | 1.5 |
| FZ-12-8 | 0.0500 | 0.0015 | 0.1416 | 0.0045 | 0.0205 | 0.0002 | 134.5 | 4.0 | 130.9 | 1.2 |
| FZ-12-9 | 0.0494 | 0.0013 | 0.1514 | 0.0043 | 0.0222 | 0.0002 | 143.2 | 3.8 | 141.5 | 1.5 |
| FZ-12-10 | 0.0481 | 0.0014 | 0.1500 | 0.0044 | 0.0228 | 0.0002 | 141.9 | 3.9 | 145.2 | 1.4 |
| FZ-12-11 | 0.0505 | 0.0025 | 0.1536 | 0.0076 | 0.0221 | 0.0003 | 145.1 | 6.7 | 140.7 | 1.9 |
| FZ-12-12 | 0.0498 | 0.0016 | 0.1521 | 0.0048 | 0.0222 | 0.0002 | 143.8 | 4.2 | 141.6 | 1.5 |
| Niutoushan Quartz monzodiorite, weighted mean ^206^Pb/^238^U age = 137.8 ± 1.8 Ma | | | | | | | | | | |
| NTS-10-1 | 0.0481 | 0.0030 | 0.1461 | 0.0094 | 0.0217 | 0.0003 | 138.5 | 8.3 | 138.4 | 2.2 |
| NTS-10-2 | 0.0547 | 0.0035 | 0.1532 | 0.0090 | 0.0209 | 0.0003 | 144.8 | 7.9 | 133.2 | 2.1 |
| NTS-10-3 | 0.0506 | 0.0028 | 0.1526 | 0.0088 | 0.0218 | 0.0004 | 144.2 | 7.7 | 138.8 | 2.3 |
| NTS-10-4 | 0.0445 | 0.0022 | 0.1331 | 0.0065 | 0.0219 | 0.0003 | 126.9 | 5.8 | 139.4 | 1.8 |
| NTS-10-5 | 0.0542 | 0.0038 | 0.1524 | 0.0096 | 0.0208 | 0.0003 | 144.1 | 8.5 | 132.7 | 2.1 |
| NTS-10-6 | 0.0495 | 0.0030 | 0.1504 | 0.0084 | 0.0225 | 0.0004 | 142.3 | 7.5 | 143.5 | 2.4 |
| NTS-10-7 | 0.0537 | 0.0033 | 0.1526 | 0.0082 | 0.0210 | 0.0003 | 144.2 | 7.3 | 133.7 | 2.1 |
| NTS-10-8 | 0.0521 | 0.0033 | 0.1537 | 0.0088 | 0.0217 | 0.0003 | 145.1 | 7.8 | 138.5 | 2.1 |
| NTS-10-9 | 0.0519 | 0.0028 | 0.1542 | 0.0078 | 0.0220 | 0.0003 | 145.6 | 6.9 | 140.0 | 2.0 |
| NTS-10-10 | 0.0515 | 0.0023 | 0.1451 | 0.0060 | 0.0208 | 0.0003 | 137.6 | 5.3 | 132.9 | 1.7 |
| NTS-10-11 | 0.0531 | 0.0026 | 0.1542 | 0.0073 | 0.0212 | 0.0003 | 145.6 | 6.4 | 135.3 | 1.6 |
| NTS-10-12 | 0.0433 | 0.0023 | 0.1386 | 0.0078 | 0.0230 | 0.0003 | 131.8 | 6.9 | 146.9 | 2.2 |
| NTS-10-13 | 0.0539 | 0.0028 | 0.1555 | 0.0072 | 0.0213 | 0.0003 | 146.7 | 6.3 | 136.1 | 1.7 |
| NTS-10-14 | 0.0492 | 0.0017 | 0.1456 | 0.0052 | 0.0214 | 0.0002 | 138.1 | 4.6 | 136.8 | 1.5 |
| NTS-10-15 | 0.0497 | 0.0016 | 0.1470 | 0.0047 | 0.0216 | 0.0003 | 139.3 | 4.2 | 137.5 | 1.6 |
| NTS-10-16 | 0.0479 | 0.0021 | 0.1423 | 0.0059 | 0.0219 | 0.0003 | 135.1 | 5.3 | 139.4 | 1.7 |
| NTS-10-17 | 0.0470 | 0.0027 | 0.1455 | 0.0078 | 0.0228 | 0.0003 | 137.9 | 6.9 | 145.2 | 2.1 |
| NTS-10-18 | 0.0476 | 0.0015 | 0.1418 | 0.0043 | 0.0217 | 0.0002 | 134.6 | 3.8 | 138.3 | 1.2 |
| Ouyangshan Quartz diorite, weighted mean^206^Pb/^238^U age = 138.4 ± 1.2 Ma | | | | | | | | | | |
| OU-32-1 | 0.0527 | 0.0034 | 0.1559 | 0.0094 | 0.0218 | 0.0004 | 147.1 | 8.3 | 138.7 | 2.3 |
| OU-32-2 | 0.0466 | 0.0020 | 0.1366 | 0.0061 | 0.0214 | 0.0002 | 130.0 | 5.5 | 136.3 | 1.5 |
| OU-32-3 | 0.0486 | 0.0022 | 0.1436 | 0.0068 | 0.0218 | 0.0003 | 136.2 | 6.0 | 138.8 | 2.2 |
| OU-32-4 | 0.0496 | 0.0031 | 0.1459 | 0.0081 | 0.0218 | 0.0004 | 138.3 | 7.2 | 139.2 | 2.3 |
| OU-32-5 | 0.0460 | 0.0022 | 0.1392 | 0.0067 | 0.0220 | 0.0002 | 132.4 | 6.0 | 140.1 | 1.6 |
| OU-32-6 | 0.0475 | 0.0027 | 0.1364 | 0.0073 | 0.0212 | 0.0003 | 129.8 | 6.6 | 135.5 | 1.7 |
| OU-32-9 | 0.0447 | 0.0022 | 0.1358 | 0.0070 | 0.0219 | 0.0003 | 129.3 | 6.3 | 139.9 | 1.7 |
| OU-32-10 | 0.0548 | 0.0029 | 0.1640 | 0.0082 | 0.0220 | 0.0003 | 154.2 | 7.2 | 140.4 | 2.2 |
| OU-32-11 | 0.0549 | 0.0037 | 0.1598 | 0.0096 | 0.0216 | 0.0004 | 150.5 | 8.4 | 138.0 | 2.6 |
| Liujiawan Quartz monzodiorite, weighted mean ^206^Pb/^238^U age = 135.0 ± 2.4 Ma | | | | | | | | | | |
| ZK12-1 | 0.0479 | 0.0023 | 0.1326 | 0.0065 | 0.0201 | 0.0003 | 126.4 | 5.8 | 128.0 | 1.6 |
| ZK12-2 | 0.0454 | 0.0020 | 0.1368 | 0.0059 | 0.0218 | 0.0003 | 130.2 | 5.2 | 139.0 | 1.8 |
| ZK12-3 | 0.0500 | 0.0015 | 0.1451 | 0.0043 | 0.0212 | 0.0003 | 137.6 | 3.8 | 135.1 | 1.8 |
| ZK12-4 | 0.0527 | 0.0018 | 0.1604 | 0.0057 | 0.0220 | 0.0003 | 151.0 | 5.0 | 140.2 | 1.8 |
| ZK12-5 | 0.0507 | 0.0029 | 0.1550 | 0.0081 | 0.0223 | 0.0003 | 146.3 | 7.1 | 141.9 | 2.2 |
| ZK12-6 | 0.0497 | 0.0020 | 0.1584 | 0.0071 | 0.0230 | 0.0003 | 149.3 | 6.2 | 146.3 | 1.8 |
| ZK12-7 | 0.0570 | 0.0022 | 0.1602 | 0.0061 | 0.0205 | 0.0003 | 150.9 | 5.3 | 130.6 | 1.7 |
| ZK12-8 | 0.0489 | 0.0020 | 0.1424 | 0.0061 | 0.0211 | 0.0003 | 135.1 | 5.4 | 134.8 | 1.9 |
| ZK12-9 | 0.0518 | 0.0026 | 0.1502 | 0.0076 | 0.0212 | 0.0003 | 142.1 | 6.7 | 135.5 | 2.1 |
| ZK12-10 | 0.0507 | 0.0021 | 0.1487 | 0.0065 | 0.0213 | 0.0003 | 140.8 | 5.7 | 135.8 | 1.8 |
| ZK12-11 | 0.0471 | 0.0019 | 0.1347 | 0.0054 | 0.0210 | 0.0003 | 128.3 | 4.8 | 134.0 | 2.1 |
| ZK12-12 | 0.0490 | 0.0019 | 0.1453 | 0.0065 | 0.0214 | 0.0004 | 137.8 | 5.8 | 136.5 | 2.4 |
| ZK12-13 | 0.0513 | 0.0022 | 0.1437 | 0.0058 | 0.0206 | 0.0003 | 136.4 | 5.2 | 131.2 | 2.0 |
| ZK12-14 | 0.0514 | 0.0025 | 0.1448 | 0.0071 | 0.0206 | 0.0003 | 137.3 | 6.3 | 131.4 | 1.9 |
| ZK12-15 | 0.0485 | 0.0018 | 0.1393 | 0.0053 | 0.0210 | 0.0003 | 132.5 | 4.7 | 133.9 | 1.8 |
| ZK12-16 | 0.0514 | 0.0022 | 0.1447 | 0.0065 | 0.0207 | 0.0003 | 137.2 | 5.8 | 131.9 | 2.1 |
| ZK12-17 | 0.0489 | 0.0019 | 0.1398 | 0.0054 | 0.0208 | 0.0003 | 132.9 | 4.8 | 133.0 | 1.7 |
| Bengqiaodi Quartz monzodiorite, weighted mean ^206^Pb/^238^U age = 138.7 ± 1.1 Ma | | | | | | | | | | |
| ZK1401-1 | 0.0451 | 0.0019 | 0.1394 | 0.0054 | 0.0220 | 0.0002 | 132.5 | 4.8 | 140.1 | 1.1 |
| ZK1401-2 | 0.0506 | 0.0019 | 0.1490 | 0.0053 | 0.0215 | 0.0002 | 141.0 | 4.7 | 137.4 | 1.3 |
| ZK1401-3 | 0.0503 | 0.0020 | 0.1534 | 0.0061 | 0.0224 | 0.0003 | 144.9 | 5.4 | 142.9 | 1.6 |
| ZK1401-4 | 0.0495 | 0.0019 | 0.1486 | 0.0058 | 0.0220 | 0.0002 | 140.6 | 5.1 | 140.2 | 1.2 |
| ZK1401-5 | 0.0528 | 0.0034 | 0.1536 | 0.0097 | 0.0216 | 0.0002 | 145.1 | 8.6 | 137.5 | 1.5 |
| ZK1401-6 | 0.0516 | 0.0018 | 0.1490 | 0.0051 | 0.0212 | 0.0002 | 141.1 | 4.5 | 135.4 | 1.2 |
| ZK1401-7 | 0.0504 | 0.0016 | 0.1490 | 0.0046 | 0.0216 | 0.0002 | 141.0 | 4.1 | 137.8 | 1.1 |
| ZK1401-8 | 0.0525 | 0.0015 | 0.1572 | 0.0044 | 0.0218 | 0.0002 | 148.3 | 3.9 | 139.2 | 1.0 |
| ZK1401-9 | 0.0522 | 0.0018 | 0.1556 | 0.0050 | 0.0218 | 0.0002 | 146.8 | 4.4 | 139.2 | 1.3 |
| ZK1401-10 | 0.0524 | 0.0024 | 0.1587 | 0.0068 | 0.0223 | 0.0002 | 149.5 | 5.9 | 142.4 | 1.5 |
| ZK1401-11 | 0.0540 | 0.0023 | 0.1606 | 0.0066 | 0.0217 | 0.0002 | 151.2 | 5.8 | 138.4 | 1.4 |
| ZK1401-12 | 0.0502 | 0.0023 | 0.1481 | 0.0063 | 0.0216 | 0.0002 | 140.2 | 5.6 | 137.5 | 1.4 |
| ZK1401-13 | 0.0528 | 0.0018 | 0.1591 | 0.0053 | 0.0219 | 0.0002 | 149.9 | 4.6 | 139.9 | 1.2 |
| ZK1401-14 | 0.0538 | 0.0018 | 0.1573 | 0.0050 | 0.0213 | 0.0002 | 148.4 | 4.4 | 136.0 | 1.2 |
| ZK1401-15 | 0.0514 | 0.0016 | 0.1571 | 0.0052 | 0.0222 | 0.0003 | 148.2 | 4.6 | 141.3 | 1.9 |

**Table 2** Major elements of apatite

| Fuzishan | | | | | | | | | | | | | | | | | | | | | | | | | | | | | | | | | | | | | | | | | | | | | | | | | | | | | | | | | | | | | | | | | | | | | | | | | | | |
| --- | --- | --- | --- | --- | --- | --- | --- | --- | --- | --- | --- | --- | --- | --- | --- | --- | --- | --- | --- | --- | --- | --- | --- | --- | --- | --- | --- | --- | --- | --- | --- | --- | --- | --- | --- | --- | --- | --- | --- | --- | --- | --- | --- | --- | --- | --- | --- | --- | --- | --- | --- | --- | --- | --- | --- | --- | --- | --- | --- | --- | --- | --- | --- | --- | --- | --- | --- | --- | --- | --- | --- | --- | --- | --- | --- |
| Chemical composition (oxide wt%) | | | | | | | | | | | | | | | | | | | | | | | | | | | | | | | | | | | | | | | | | | | | | | | | | | | | | | | | | | | | | | | | | | | | | | | | | | | |
| SiO_2_ | 0.40 | | 0.48 | | | | | | 0.38 | 0.37 | | | | | | 0.30 | | | | | 0.42 | | | | | | 0.39 | | | | | 0.20 | | 0.28 | | | | 0.45 | | | | | 0.13 | | | | | | 0.31 | | | | 0.41 | | | 0.25 | | | | | 0.15 | | | | 0.17 | | | 0.36 | | 0.25 | | | | 0.34 | |
| FeO | 0.07 | | 0.03 | | | | | | 0.01 | 0.03 | | | | | | 0.09 | | | | | 0.05 | | | | | | 0.09 | | | | | b.d.l | | 0.07 | | | | 0.05 | | | | | 0.02 | | | | | | 0.04 | | | | b.d.l | | | 0.03 | | | | | 0.02 | | | | 0.06 | | | b.d.l | | 0.06 | | | | 0.03 | |
| CaO | 54.11 | | 54.48 | | | | | | 54.24 | 53.99 | | | | | | 54.18 | | | | | 54.05 | | | | | | 54.10 | | | | | 54.16 | | 54.13 | | | | 53.54 | | | | | 53.80 | | | | | | 53.50 | | | | 53.51 | | | 53.73 | | | | | 53.19 | | | | 53.63 | | | 53.35 | | 53.42 | | | | 51.37 | |
| Na_2_O | 0.06 | | 0.08 | | | | | | 0.10 | 0.09 | | | | | | 0.02 | | | | | 0.15 | | | | | | 0.15 | | | | | 0.02 | | 0.17 | | | | 0.01 | | | | | 0.05 | | | | | | 0.05 | | | | 0.07 | | | 0.13 | | | | | 0.09 | | | | 0.07 | | | 0.03 | | 0.02 | | | | 0.06 | |
| P_2_O_5_ | 41.21 | | 41.19 | | | | | | 41.57 | 42.37 | | | | | | 41.56 | | | | | 41.47 | | | | | | 40.89 | | | | | 42.26 | | 41.51 | | | | 40.81 | | | | | 42.07 | | | | | | 41.04 | | | | 40.66 | | | 41.35 | | | | | 41.09 | | | | 41.56 | | | 41.38 | | 40.41 | | | | 42.26 | |
| MgO | 0.01 | | 0.01 | | | | | | b.d.l | b.d.l | | | | | | b.d.l | | | | | 0.01 | | | | | | 0.02 | | | | | b.d.l | | 0.01 | | | | 0.04 | | | | | 0.03 | | | | | | 0.02 | | | | 0.00 | | | 0.02 | | | | | 0.02 | | | | 0.01 | | | 0.01 | | b.d.l | | | | 0.02 | |
| MnO | 0.14 | | 0.04 | | | | | | 0.13 | 0.13 | | | | | | 0.03 | | | | | 0.09 | | | | | | 0.10 | | | | | 0.06 | | 0.18 | | | | 0.06 | | | | | 0.05 | | | | | | 0.12 | | | | 0.08 | | | 0.01 | | | | | 0.09 | | | | 0.08 | | | 0.12 | | 0.02 | | | | 0.06 | |
| SO_3_ | 0.46 | | 0.45 | | | | | | 0.35 | 0.44 | | | | | | 0.08 | | | | | 0.71 | | | | | | 0.52 | | | | | 0.09 | | 0.62 | | | | 0.28 | | | | | 0.12 | | | | | | 0.18 | | | | 0.28 | | | 0.47 | | | | | 0.14 | | | | 0.15 | | | 0.20 | | 0.18 | | | | 0.12 | |
| F | 2.20 | | 2.97 | | | | | | 2.93 | 2.51 | | | | | | 2.50 | | | | | 2.23 | | | | | | 2.19 | | | | | 2.72 | | 3.18 | | | | 2.55 | | | | | 2.47 | | | | | | 2.43 | | | | 2.74 | | | 2.21 | | | | | 2.05 | | | | 2.65 | | | 2.72 | | 2.30 | | | | 1.49 | |
| Cl | 0.37 | | 0.36 | | | | | | 0.34 | 0.40 | | | | | | 0.34 | | | | | 0.37 | | | | | | 0.33 | | | | | 0.35 | | 0.31 | | | | 0.35 | | | | | 0.19 | | | | | | 0.36 | | | | 0.25 | | | 0.44 | | | | | 0.28 | | | | 0.31 | | | 0.32 | | 0.31 | | | | 0.30 | |
| Sub-Total | 99.02 | | 100.08 | | | | | | 100.04 | 100.32 | | | | | | 99.10 | | | | | 99.53 | | | | | | 98.79 | | | | | 99.86 | | 100.45 | | | | 98.13 | | | | | 98.92 | | | | | | 98.04 | | | | 98.01 | | | 98.63 | | | | | 97.12 | | | | 98.69 | | | 98.49 | | 96.98 | | | | 96.04 | |
| O=F+Cl | 1.01 | | 1.33 | | | | | | 1.31 | 1.15 | | | | | | 1.13 | | | | | 1.02 | | | | | | 1.00 | | | | | 1.23 | | 1.41 | | | | 1.15 | | | | | 1.08 | | | | | | 1.10 | | | | 1.21 | | | 1.03 | | | | | 0.93 | | | | 1.18 | | | 1.22 | | 1.04 | | | | 0.69 | |
| Total | 98.01 | | 98.75 | | | | | | 98.73 | 99.18 | | | | | | 97.97 | | | | | 98.51 | | | | | | 97.79 | | | | | 98.64 | | 99.05 | | | | 96.98 | | | | | 97.84 | | | | | | 96.94 | | | | 96.80 | | | 97.61 | | | | | 96.19 | | | | 97.51 | | | 97.27 | | 95.94 | | | | 95.34 | |
| Si | 0.034 | | 0.041 | | | | | | 0.032 | 0.031 | | | | | | 0.026 | | | | | 0.036 | | | | | | 0.034 | | | | | 0.017 | | 0.024 | | | | 0.039 | | | | | 0.011 | | | | | | 0.027 | | | | 0.035 | | | 0.021 | | | | | 0.013 | | | | 0.015 | | | 0.031 | | 0.022 | | | | 0.030 | |
| Fe | 0.005 | | 0.002 | | | | | | 0.001 | 0.002 | | | | | | 0.006 | | | | | 0.003 | | | | | | 0.006 | | | | |  | | 0.005 | | | | 0.003 | | | | | 0.001 | | | | | | 0.003 | | | |  | | | 0.002 | | | | | 0.001 | | | | 0.004 | | |  | | 0.004 | | | | 0.002 | |
| Ca | 4.939 | | 4.952 | | | | | | 4.932 | 4.886 | | | | | | 4.954 | | | | | 4.909 | | | | | | 4.941 | | | | | 4.930 | | 4.914 | | | | 4.948 | | | | | 4.923 | | | | | | 4.943 | | | | 4.955 | | | 4.926 | | | | | 4.939 | | | | 4.932 | | | 4.924 | | 4.980 | | | | 4.815 | |
| Na | 0.009 | | 0.013 | | | | | | 0.016 | 0.015 | | | | | | 0.004 | | | | | 0.024 | | | | | | 0.024 | | | | | 0.003 | | 0.027 | | | | 0.002 | | | | | 0.009 | | | | | | 0.009 | | | | 0.011 | | | 0.021 | | | | | 0.015 | | | | 0.012 | | | 0.005 | | 0.004 | | | | 0.009 | |
| P | 2.972 | | 2.959 | | | | | | 2.987 | 3.029 | | | | | | 3.003 | | | | | 2.976 | | | | | | 2.951 | | | | | 3.040 | | 2.977 | | | | 2.981 | | | | | 3.042 | | | | | | 2.996 | | | | 2.975 | | | 2.996 | | | | | 3.014 | | | | 3.020 | | | 3.017 | | 2.977 | | | | 3.129 | |
| Mg | 0.002 | | 0.002 | | | | | |  |  | | | | | |  | | | | | 0.001 | | | | | | 0.003 | | | | |  | | 0.001 | | | | 0.005 | | | | | 0.003 | | | | | | 0.003 | | | | 0.000 | | | 0.002 | | | | | 0.003 | | | | 0.001 | | | 0.001 | |  | | | | 0.003 | |
| S | 0.029 | | 0.029 | | | | | | 0.022 | 0.028 | | | | | | 0.005 | | | | | 0.045 | | | | | | 0.033 | | | | | 0.006 | | 0.040 | | | | 0.018 | | | | | 0.007 | | | | | | 0.012 | | | | 0.018 | | | 0.030 | | | | | 0.009 | | | | 0.009 | | | 0.013 | | 0.012 | | | | 0.008 | |
| Mn | 0.010 | | 0.003 | | | | | | 0.009 | 0.010 | | | | | | 0.002 | | | | | 0.007 | | | | | | 0.007 | | | | | 0.004 | | 0.013 | | | | 0.004 | | | | | 0.004 | | | | | | 0.008 | | | | 0.006 | | | 0.001 | | | | | 0.007 | | | | 0.006 | | | 0.009 | | 0.002 | | | | 0.004 | |
| F | 0.585 | | 0.788 | | | | | | 0.778 | 0.667 | | | | | | 0.663 | | | | | 0.592 | | | | | | 0.581 | | | | | 0.723 | | 0.843 | | | | 0.677 | | | | | 0.656 | | | | | | 0.644 | | | | 0.728 | | | 0.586 | | | | | 0.544 | | | | 0.703 | | | 0.723 | | 0.611 | | | | 0.395 | |
| Cl | 0.054 | | 0.053 | | | | | | 0.050 | 0.059 | | | | | | 0.051 | | | | | 0.054 | | | | | | 0.048 | | | | | 0.052 | | 0.046 | | | | 0.052 | | | | | 0.028 | | | | | | 0.053 | | | | 0.037 | | | 0.065 | | | | | 0.041 | | | | 0.045 | | | 0.047 | | 0.045 | | | | 0.044 | |
| OH | 0.361 | | 0.159 | | | | | | 0.173 | 0.274 | | | | | | 0.287 | | | | | 0.355 | | | | | | 0.371 | | | | | 0.226 | | 0.111 | | | | 0.272 | | | | | 0.316 | | | | | | 0.303 | | | | 0.234 | | | 0.350 | | | | | 0.414 | | | | 0.252 | | | 0.230 | | 0.343 | | | | 0.561 | |
| Niutoushan | | | | | | | | | | | | | | | | | | | | | | | | | | | | | | | | | | | | | | | | | | | | | | | | | | | | | | | | | | | | | | | | | | | | | | | | | | | |
| Chemical composition (oxide wt%) | | | | | | | | | | | | | | | | | | | | | | | | | | | | | | | | | | | | | | | | | | | | | | | | | | | | | | | | | | | | | | | | | | | | | | | | | | | |
| SiO_2_ | | 0.31 | | | 0.25 | | | | | | | 0.37 | | | | | 0.35 | | | | | | 0.30 | | | | | 0.29 | | | | | | 0.24 | | | | | 0.31 | | | | | | 0.31 | | | | | 0.33 | | | | | | | 0.35 | | | | | 0.24 | | | | 0.14 | | | | | 0.26 | | | | 0.55 |
| FeO | | 0.04 | | | 0.04 | | | | | | | 0.05 | | | | | 0.05 | | | | | | 0.05 | | | | | 0.04 | | | | | | 0.05 | | | | | 0.08 | | | | | | 0.09 | | | | | 0.07 | | | | | | | 0.09 | | | | | 0.03 | | | | 0.03 | | | | | 0.01 | | | | 0.06 |
| CaO | | 54.58 | | | 54.41 | | | | | | | 54.40 | | | | | 54.52 | | | | | | 54.71 | | | | | 54.21 | | | | | | 54.30 | | | | | 54.57 | | | | | | 53.96 | | | | | 53.61 | | | | | | | 53.45 | | | | | 53.43 | | | | 53.57 | | | | | 53.53 | | | | 53.09 |
| Na_2_O | | 0.02 | | | 0.06 | | | | | | | 0.04 | | | | | 0.01 | | | | | | 0.01 | | | | | 0.02 | | | | | | 0.03 | | | | | 0.04 | | | | | | 0.08 | | | | | 0.11 | | | | | | | 0.01 | | | | | 0.05 | | | | 0.02 | | | | | 0.04 | | | | 0.02 |
| P_2_O_5_ | | 41.48 | | | 41.78 | | | | | | | 41.44 | | | | | 41.75 | | | | | | 40.29 | | | | | 41.98 | | | | | | 41.80 | | | | | 42.06 | | | | | | 41.22 | | | | | 41.40 | | | | | | | 41.60 | | | | | 41.68 | | | | 41.31 | | | | | 41.13 | | | | 40.92 |
| MgO | | 0.01 | | | b.d.l | | | | | | | 0.01 | | | | | 0.02 | | | | | | 0.01 | | | | | 0.04 | | | | | | 0.01 | | | | | b.d.l | | | | | | 0.01 | | | | | 0.01 | | | | | | | 0.00 | | | | | b.d.l | | | | b.d.l | | | | | 0.01 | | | | 0.02 |
| MnO | | 0.15 | | | 0.17 | | | | | | | 0.04 | | | | | 0.13 | | | | | | 0.13 | | | | | 0.11 | | | | | | 0.09 | | | | | 0.09 | | | | | | 0.08 | | | | | 0.16 | | | | | | | 0.07 | | | | | 0.06 | | | | 0.16 | | | | | 0.09 | | | | 0.09 |
| SO_3_ | | 0.24 | | | 0.18 | | | | | | | 0.24 | | | | | 0.27 | | | | | | 0.32 | | | | | 0.21 | | | | | | 0.23 | | | | | 0.17 | | | | | | 0.48 | | | | | 0.31 | | | | | | | 0.22 | | | | | 0.23 | | | | 0.18 | | | | | 0.25 | | | | 0.20 |
| F | | 2.22 | | | 2.69 | | | | | | | 1.84 | | | | | 1.92 | | | | | | 2.18 | | | | | 2.19 | | | | | | 2.61 | | | | | 2.29 | | | | | | 1.98 | | | | | 1.80 | | | | | | | 2.09 | | | | | 2.27 | | | | 2.53 | | | | | 2.33 | | | | 2.12 |
| Cl | | 0.53 | | | 0.44 | | | | | | | 0.43 | | | | | 0.50 | | | | | | 0.56 | | | | | 0.48 | | | | | | 0.57 | | | | | 0.45 | | | | | | 0.56 | | | | | 0.44 | | | | | | | 0.51 | | | | | 0.42 | | | | 0.28 | | | | | 0.33 | | | | 0.52 |
| Sub-Total | | 99.58 | | | 100.01 | | | | | | | 98.87 | | | | | 99.52 | | | | | | 98.56 | | | | | 99.56 | | | | | | 99.93 | | | | | 100.06 | | | | | | 98.76 | | | | | 98.24 | | | | | | | 98.40 | | | | | 98.40 | | | | 98.20 | | | | | 97.99 | | | | 97.58 |
| O=F+Cl | | 1.05 | | | 1.23 | | | | | | | 0.87 | | | | | 0.92 | | | | | | 1.04 | | | | | 1.03 | | | | | | 1.23 | | | | | 1.07 | | | | | | 0.96 | | | | | 0.86 | | | | | | | 1.00 | | | | | 1.05 | | | | 1.13 | | | | | 1.06 | | | | 1.01 |
| Total | | 98.52 | | | 98.78 | | | | | | | 97.99 | | | | | 98.60 | | | | | | 97.51 | | | | | 98.54 | | | | | | 98.70 | | | | | 98.99 | | | | | | 97.79 | | | | | 97.38 | | | | | | | 97.40 | | | | | 97.35 | | | | 97.08 | | | | | 96.93 | | | | 96.57 |
| Si | | 0.027 | | | 0.021 | | | | | | | 0.031 | | | | | 0.030 | | | | | | 0.025 | | | | | 0.024 | | | | | | 0.020 | | | | | 0.026 | | | | | | 0.027 | | | | | 0.028 | | | | | | | 0.030 | | | | | 0.021 | | | | 0.012 | | | | | 0.022 | | | | 0.047 |
| Fe | | 0.003 | | | 0.003 | | | | | | | 0.004 | | | | | 0.004 | | | | | | 0.004 | | | | | 0.003 | | | | | | 0.004 | | | | | 0.005 | | | | | | 0.006 | | | | | 0.005 | | | | | | | 0.006 | | | | | 0.002 | | | | 0.002 | | | | | 0.001 | | | | 0.004 |
| Ca | | 4.961 | | | 4.944 | | | | | | | 4.956 | | | | | 4.945 | | | | | | 5.018 | | | | | 4.928 | | | | | | 4.943 | | | | | 4.937 | | | | | | 4.937 | | | | | 4.916 | | | | | | | 4.918 | | | | | 4.918 | | | | 4.946 | | | | | 4.944 | | | | 4.924 |
| Na | | 0.003 | | | 0.009 | | | | | | | 0.006 | | | | | 0.001 | | | | | | 0.002 | | | | | 0.004 | | | | | | 0.005 | | | | | 0.007 | | | | | | 0.013 | | | | | 0.019 | | | | | | | 0.002 | | | | | 0.008 | | | | 0.003 | | | | | 0.007 | | | | 0.003 |
| P | | 2.979 | | | 2.999 | | | | | | | 2.983 | | | | | 2.991 | | | | | | 2.919 | | | | | 3.015 | | | | | | 3.006 | | | | | 3.007 | | | | | | 2.980 | | | | | 3.000 | | | | | | | 3.024 | | | | | 3.032 | | | | 3.014 | | | | | 3.002 | | | | 2.999 |
| Mg | | 0.001 | | | b.d.l | | | | | | | 0.001 | | | | | 0.002 | | | | | | 0.002 | | | | | 0.005 | | | | | | 0.001 | | | | | b.d.l | | | | | | 0.001 | | | | | 0.001 | | | | | | | 0.000 | | | | | b.d.l | | | | b.d.l | | | | | 0.001 | | | | 0.003 |
| S | | 0.015 | | | 0.012 | | | | | | | 0.016 | | | | | 0.017 | | | | | | 0.020 | | | | | 0.014 | | | | | | 0.015 | | | | | 0.011 | | | | | | 0.030 | | | | | 0.020 | | | | | | | 0.014 | | | | | 0.015 | | | | 0.012 | | | | | 0.016 | | | | 0.013 |
| Mn | | 0.011 | | | 0.012 | | | | | | | 0.003 | | | | | 0.010 | | | | | | 0.009 | | | | | 0.008 | | | | | | 0.006 | | | | | 0.007 | | | | | | 0.006 | | | | | 0.011 | | | | | | | 0.005 | | | | | 0.004 | | | | 0.011 | | | | | 0.007 | | | | 0.006 |
| F | | 0.590 | | | 0.713 | | | | | | | 0.488 | | | | | 0.508 | | | | | | 0.579 | | | | | 0.581 | | | | | | 0.693 | | | | | 0.607 | | | | | | 0.526 | | | | | 0.477 | | | | | | | 0.555 | | | | | 0.602 | | | | 0.671 | | | | | 0.620 | | | | 0.561 |
| Cl | | 0.077 | | | 0.065 | | | | | | | 0.064 | | | | | 0.073 | | | | | | 0.082 | | | | | 0.070 | | | | | | 0.084 | | | | | 0.066 | | | | | | 0.082 | | | | | 0.065 | | | | | | | 0.075 | | | | | 0.062 | | | | 0.041 | | | | | 0.048 | | | | 0.077 |
| OH | | 0.333 | | | 0.222 | | | | | | | 0.448 | | | | | 0.418 | | | | | | 0.339 | | | | | 0.350 | | | | | | 0.223 | | | | | 0.326 | | | | | | 0.392 | | | | | 0.459 | | | | | | | 0.370 | | | | | 0.336 | | | | 0.288 | | | | | 0.332 | | | | 0.362 |
| Ouyangshan | | | | | | | | | | | | | | | | | | | | | | | | | | | | | | | | | | | | | | | | | | | | | | | | | | | | | | | | | | | | | | | | | | | | | | | | | | | |
| Chemical composition (oxide wt%) | | | | | | | | | | | | | | | | | | | | | | | | | | | | | | | | | | | | | | | | | | | | | | | | | | | | | | | | | | | | | | | | | | | | | | | | | | | |
| SiO_2_ | | 0.55 | | | | | 0.49 | | | | | | 0.64 | | | | | 0.40 | | | | | | 0.29 | | | | | | 0.36 | | | | | 0.19 | | | | | 0.45 | | | | | | 0.30 | | | | | 0.25 | | | | | | | 0.27 | | | | | 0.24 | | | | 0.26 | | | | | 0.27 | | | 0.27 |
| FeO | | b.d.l | | | | | 0.05 | | | | | | 0.01 | | | | | 0.01 | | | | | | 0.01 | | | | | | b.d.l | | | | | 0.02 | | | | | 0.05 | | | | | | 0.01 | | | | | 0.11 | | | | | | | b.d.l | | | | | 0.02 | | | | 0.01 | | | | | 0.02 | | | 0.07 |
| CaO | | 53.52 | | | | | 53.50 | | | | | | 53.76 | | | | | 53.32 | | | | | | 54.53 | | | | | | 53.68 | | | | | 54.11 | | | | | 53.56 | | | | | | 53.75 | | | | | 53.71 | | | | | | | 53.54 | | | | | 53.89 | | | | 53.34 | | | | | 53.56 | | | 53.76 |
| Na_2_O | | 0.12 | | | | | 0.06 | | | | | | 0.04 | | | | | 0.12 | | | | | | 0.12 | | | | | | 0.08 | | | | | 0.03 | | | | | 0.08 | | | | | | 0.13 | | | | | 0.09 | | | | | | | 0.24 | | | | | 0.13 | | | | 0.22 | | | | | 0.19 | | | 0.13 |
| P_2_O_5_ | | 40.73 | | | | | 40.91 | | | | | | 40.32 | | | | | 41.70 | | | | | | 41.21 | | | | | | 41.15 | | | | | 41.53 | | | | | 41.16 | | | | | | 40.81 | | | | | 41.50 | | | | | | | 41.83 | | | | | 41.36 | | | | 40.79 | | | | | 40.91 | | | 41.87 |
| MgO | | b.d.l | | | | | 0.02 | | | | | | b.d.l | | | | | 0.02 | | | | | | b.d.l | | | | | | b.d.l | | | | | 0.00 | | | | | 0.02 | | | | | | b.d.l | | | | | 0.03 | | | | | | | 0.00 | | | | | 0.01 | | | | b.d.l | | | | | b.d.l | | | b.d.l |
| MnO | | 0.07 | | | | | 0.04 | | | | | | 0.14 | | | | | 0.02 | | | | | | 0.09 | | | | | | 0.02 | | | | | 0.08 | | | | | 0.11 | | | | | | 0.14 | | | | | 0.11 | | | | | | | 0.06 | | | | | 0.04 | | | | 0.07 | | | | | 0.03 | | | 0.06 |
| SO_3_ | | 0.44 | | | | | 0.49 | | | | | | 0.23 | | | | | 0.53 | | | | | | 0.39 | | | | | | 0.41 | | | | | 0.21 | | | | | 0.43 | | | | | | 0.64 | | | | | 0.48 | | | | | | | 0.18 | | | | | 0.47 | | | | 0.48 | | | | | 0.57 | | | 0.45 |
| F | | 2.97 | | | | | 1.64 | | | | | | 2.22 | | | | | 2.07 | | | | | | 2.49 | | | | | | 2.48 | | | | | 2.40 | | | | | 2.23 | | | | | | 2.14 | | | | | 2.48 | | | | | | | 2.32 | | | | | 2.22 | | | | 1.98 | | | | | 2.27 | | | 2.11 |
| Cl | | 0.25 | | | | | 0.26 | | | | | | 0.31 | | | | | 0.27 | | | | | | 0.26 | | | | | | 0.23 | | | | | 0.22 | | | | | 0.37 | | | | | | 0.29 | | | | | 0.20 | | | | | | | 0.28 | | | | | 0.32 | | | | 0.22 | | | | | 0.30 | | | 0.25 |
| Sub-Total | | 98.65 | | | | | 97.46 | | | | | | 97.68 | | | | | 98.46 | | | | | | 99.38 | | | | | | 98.41 | | | | | 98.78 | | | | | 98.47 | | | | | | 98.20 | | | | | 98.95 | | | | | | | 98.72 | | | | | 98.68 | | | | 97.38 | | | | | 98.12 | | | 98.96 |
| O=F+Cl | | 1.31 | | | | | 0.75 | | | | | | 1.01 | | | | | 0.93 | | | | | | 1.11 | | | | | | 1.09 | | | | | 1.06 | | | | | 1.02 | | | | | | 0.97 | | | | | 1.09 | | | | | | | 1.04 | | | | | 1.01 | | | | 0.88 | | | | | 1.03 | | | 0.94 |
| Total | | 97.34 | | | | | 96.71 | | | | | | 96.67 | | | | | 97.53 | | | | | | 98.27 | | | | | | 97.31 | | | | | 97.72 | | | | | 97.44 | | | | | | 97.24 | | | | | 97.87 | | | | | | | 97.68 | | | | | 97.67 | | | | 96.49 | | | | | 97.10 | | | 98.02 |
| Si | | 0.047 | | | | | 0.042 | | | | | | 0.055 | | | | | 0.034 | | | | | | 0.025 | | | | | | 0.031 | | | | | 0.016 | | | | | 0.039 | | | | | | 0.026 | | | | | 0.021 | | | | | | | 0.023 | | | | | 0.021 | | | | 0.023 | | | | | 0.023 | | | 0.023 |
| Fe | |  | | | | | 0.003 | | | | | | 0.001 | | | | | 0.000 | | | | | | 0.001 | | | | | |  | | | | | 0.002 | | | | | 0.004 | | | | | | 0.001 | | | | | 0.007 | | | | | | |  | | | | | 0.001 | | | | 0.001 | | | | | 0.001 | | | 0.005 |
| Ca | | 4.933 | | | | | 4.929 | | | | | | 4.968 | | | | | 4.887 | | | | | | 4.961 | | | | | | 4.938 | | | | | 4.954 | | | | | 4.919 | | | | | | 4.939 | | | | | 4.914 | | | | | | | 4.897 | | | | | 4.932 | | | | 4.926 | | | | | 4.930 | | | 4.901 |
| Na | | 0.020 | | | | | 0.010 | | | | | | 0.006 | | | | | 0.020 | | | | | | 0.019 | | | | | | 0.013 | | | | | 0.004 | | | | | 0.014 | | | | | | 0.021 | | | | | 0.015 | | | | | | | 0.040 | | | | | 0.022 | | | | 0.037 | | | | | 0.031 | | | 0.021 |
| P | | 2.966 | | | | | 2.979 | | | | | | 2.944 | | | | | 3.020 | | | | | | 2.963 | | | | | | 2.991 | | | | | 3.005 | | | | | 2.987 | | | | | | 2.963 | | | | | 3.000 | | | | | | | 3.024 | | | | | 2.991 | | | | 2.977 | | | | | 2.975 | | | 3.017 |
| Mg | |  | | | | | 0.003 | | | | | |  | | | | | 0.003 | | | | | |  | | | | | |  | | | | | 0.001 | | | | | 0.003 | | | | | |  | | | | | 0.004 | | | | | | | 0.001 | | | | | 0.001 | | | |  | | | | |  | | |  |
| S | | 0.029 | | | | | 0.032 | | | | | | 0.015 | | | | | 0.034 | | | | | | 0.025 | | | | | | 0.027 | | | | | 0.013 | | | | | 0.027 | | | | | | 0.041 | | | | | 0.031 | | | | | | | 0.012 | | | | | 0.030 | | | | 0.031 | | | | | 0.037 | | | 0.029 |
| Mn | | 0.005 | | | | | 0.003 | | | | | | 0.011 | | | | | 0.002 | | | | | | 0.006 | | | | | | 0.001 | | | | | 0.006 | | | | | 0.008 | | | | | | 0.010 | | | | | 0.008 | | | | | | | 0.004 | | | | | 0.003 | | | | 0.005 | | | | | 0.002 | | | 0.005 |
| F | | 0.789 | | | | | 0.435 | | | | | | 0.590 | | | | | 0.549 | | | | | | 0.661 | | | | | | 0.657 | | | | | 0.637 | | | | | 0.593 | | | | | | 0.568 | | | | | 0.658 | | | | | | | 0.616 | | | | | 0.589 | | | | 0.527 | | | | | 0.604 | | | 0.560 |
| Cl | | 0.037 | | | | | 0.038 | | | | | | 0.046 | | | | | 0.039 | | | | | | 0.038 | | | | | | 0.034 | | | | | 0.032 | | | | | 0.055 | | | | | | 0.043 | | | | | 0.029 | | | | | | | 0.041 | | | | | 0.047 | | | | 0.032 | | | | | 0.044 | | | 0.037 |
| OH | | 0.174 | | | | | 0.527 | | | | | | 0.364 | | | | | 0.412 | | | | | | 0.301 | | | | | | 0.309 | | | | | 0.331 | | | | | 0.353 | | | | | | 0.390 | | | | | 0.313 | | | | | | | 0.343 | | | | | 0.364 | | | | 0.441 | | | | | 0.352 | | | 0.404 |
| Liujiawan | | | | | | | | | | | | | | | | | | | | | | | | | | | | | | | | | | | | | | | | | | | | | | | | | | | | | | | | | | | | | | | | | | | | | | | | | | | |
| Chemical composition (oxide wt%) | | | | | | | | | | | | | | | | | | | | | | | | | | | | | | | | | | | | | | | | | | | | | | | | | | | | | | | | | | | | | | | | | | | | | | | | | | | |
| SiO_2_ | | 0.18 | | | | 0.23 | | | | | | | | 0.18 | | | | | | 0.22 | | | | | 0.23 | | | | | | 0.19 | | | | | 0.15 | | | | | | 0.24 | | | | | 0.24 | | | | | | | 0.23 | | | | | | 0.24 | | | | 0.19 | | | | | 0.22 | | | | 0.21 | | |
| FeO | | 0.08 | | | | b.d.l | | | | | | | | b.d.l | | | | | | 0.01 | | | | | b.d.l | | | | | | b.d.l | | | | | b.d.l | | | | | | 0.03 | | | | | b.d.l | | | | | | | b.d.l | | | | | | 0.08 | | | | 0.05 | | | | | b.d.l | | | | 0.10 | | |
| CaO | | 54.45 | | | | 54.50 | | | | | | | | 54.55 | | | | | | 54.05 | | | | | 54.89 | | | | | | 53.10 | | | | | 54.42 | | | | | | 54.37 | | | | | 53.64 | | | | | | | 53.63 | | | | | | 54.33 | | | | 54.12 | | | | | 54.38 | | | | 54.37 | | |
| Na_2_O | | 0.06 | | | | 0.04 | | | | | | | | 0.05 | | | | | | 0.09 | | | | | 0.04 | | | | | | 0.08 | | | | | 0.04 | | | | | | 0.02 | | | | | 0.04 | | | | | | | 0.03 | | | | | | 0.10 | | | | 0.10 | | | | | 0.08 | | | | 0.08 | | |
| P_2_O_5_ | | 42.12 | | | | 41.71 | | | | | | | | 42.50 | | | | | | 42.07 | | | | | 42.34 | | | | | | 41.21 | | | | | 41.91 | | | | | | 41.72 | | | | | 41.98 | | | | | | | 41.48 | | | | | | 41.25 | | | | 41.63 | | | | | 42.33 | | | | 40.83 | | |
| MgO | | 0.02 | | | | 0.02 | | | | | | | | 0.02 | | | | | | b.d.l | | | | | b.d.l | | | | | | 0.01 | | | | | 0.01 | | | | | | 0.01 | | | | | 0.02 | | | | | | | 0.03 | | | | | | 0.00 | | | | 0.04 | | | | | 0.01 | | | | 0.03 | | |
| MnO | | 0.09 | | | | 0.12 | | | | | | | | 0.04 | | | | | | 0.07 | | | | | 0.06 | | | | | | 0.08 | | | | | 0.07 | | | | | | 0.08 | | | | | 0.03 | | | | | | | 0.10 | | | | | | 0.07 | | | | 0.08 | | | | | 0.03 | | | | 0.04 | | |
| SO_3_ | | 0.11 | | | | 0.13 | | | | | | | | 0.20 | | | | | | 0.21 | | | | | 0.23 | | | | | | 0.12 | | | | | 0.18 | | | | | | 0.19 | | | | | 0.16 | | | | | | | 0.19 | | | | | | 0.21 | | | | 0.20 | | | | | 0.12 | | | | 0.16 | | |
| F | | 2.19 | | | | 3.75 | | | | | | | | 2.08 | | | | | | 2.79 | | | | | 2.75 | | | | | | 3.83 | | | | | 2.99 | | | | | | 3.00 | | | | | 4.23 | | | | | | | 3.11 | | | | | | 2.26 | | | | 2.54 | | | | | 3.10 | | | | 3.39 | | |
| Cl | | 0.17 | | | | 0.15 | | | | | | | | 0.12 | | | | | | 0.09 | | | | | 0.11 | | | | | | 0.17 | | | | | 0.10 | | | | | | 0.14 | | | | | 0.10 | | | | | | | 0.31 | | | | | | 0.12 | | | | 0.29 | | | | | 0.15 | | | | 0.13 | | |
| Sub-Total | | 99.49 | | | | 100.66 | | | | | | | | 99.73 | | | | | | 99.58 | | | | | 100.63 | | | | | | 98.80 | | | | | 99.87 | | | | | | 99.82 | | | | | 100.42 | | | | | | | 99.11 | | | | | | 98.67 | | | | 99.23 | | | | | 100.44 | | | | 99.34 | | |
| O=F+Cl | | 0.96 | | | | 1.61 | | | | | | | | 0.90 | | | | | | 1.19 | | | | | 1.18 | | | | | | 1.65 | | | | | 1.28 | | | | | | 1.29 | | | | | 1.80 | | | | | | | 1.38 | | | | | | 0.98 | | | | 1.14 | | | | | 1.34 | | | | 1.46 | | |
| Total | | 98.52 | | | | 99.04 | | | | | | | | 98.82 | | | | | | 98.39 | | | | | 99.45 | | | | | | 97.15 | | | | | 98.59 | | | | | | 98.52 | | | | | 98.62 | | | | | | | 97.73 | | | | | | 97.69 | | | | 98.09 | | | | | 99.10 | | | | 97.88 | | |
| Si | | 0.015 | | | | 0.020 | | | | | | | | 0.015 | | | | | | 0.019 | | | | | 0.019 | | | | | | 0.017 | | | | | 0.013 | | | | | | 0.021 | | | | | 0.020 | | | | | | | 0.020 | | | | | | 0.020 | | | | 0.016 | | | | | 0.019 | | | | 0.018 | | |
| Fe | | 0.006 | | | |  | | | | | | | |  | | | | | | 0.001 | | | | |  | | | | | |  | | | | |  | | | | | | 0.002 | | | | |  | | | | | | |  | | | | | | 0.006 | | | | 0.003 | | | | |  | | | | 0.007 | | |
| Ca | | 4.936 | | | | 4.957 | | | | | | | | 4.927 | | | | | | 4.921 | | | | | 4.944 | | | | | | 4.931 | | | | | 4.950 | | | | | | 4.952 | | | | | 4.918 | | | | | | | 4.936 | | | | | | 4.961 | | | | 4.940 | | | | | 4.926 | | | | 4.986 | | |
| Na | | 0.010 | | | | 0.007 | | | | | | | | 0.008 | | | | | | 0.014 | | | | | 0.006 | | | | | | 0.014 | | | | | 0.007 | | | | | | 0.004 | | | | | 0.006 | | | | | | | 0.005 | | | | | | 0.017 | | | | 0.016 | | | | | 0.014 | | | | 0.013 | | |
| P | | 3.017 | | | | 2.997 | | | | | | | | 3.033 | | | | | | 3.026 | | | | | 3.013 | | | | | | 3.023 | | | | | 3.012 | | | | | | 3.002 | | | | | 3.041 | | | | | | | 3.016 | | | | | | 2.977 | | | | 3.002 | | | | | 3.030 | | | | 2.959 | | |
| Mg | | 0.003 | | | | 0.003 | | | | | | | | 0.002 | | | | | |  | | | | |  | | | | | | 0.001 | | | | | 0.002 | | | | | | 0.001 | | | | | 0.002 | | | | | | | 0.004 | | | | | | 0.000 | | | | 0.004 | | | | | 0.002 | | | | 0.004 | | |
| S | | 0.007 | | | | 0.008 | | | | | | | | 0.012 | | | | | | 0.013 | | | | | 0.014 | | | | | | 0.008 | | | | | 0.011 | | | | | | 0.012 | | | | | 0.010 | | | | | | | 0.012 | | | | | | 0.014 | | | | 0.013 | | | | | 0.008 | | | | 0.010 | | |
| Mn | | 0.007 | | | | 0.008 | | | | | | | | 0.003 | | | | | | 0.005 | | | | | 0.004 | | | | | | 0.006 | | | | | 0.005 | | | | | | 0.006 | | | | | 0.002 | | | | | | | 0.007 | | | | | | 0.005 | | | | 0.006 | | | | | 0.002 | | | | 0.003 | | |
| F | | 0.582 | | | | 0.995 | | | | | | | | 0.552 | | | | | | 0.740 | | | | | 0.729 | | | | | | 1.016 | | | | | 0.792 | | | | | | 0.796 | | | | | 1.123 | | | | | | | 0.827 | | | | | | 0.601 | | | | 0.675 | | | | | 0.823 | | | | 0.901 | | |
| Cl | | 0.025 | | | | 0.022 | | | | | | | | 0.018 | | | | | | 0.012 | | | | | 0.016 | | | | | | 0.025 | | | | | 0.015 | | | | | | 0.021 | | | | | 0.014 | | | | | | | 0.045 | | | | | | 0.018 | | | | 0.043 | | | | | 0.022 | | | | 0.019 | | |
| OH | | 0.392 | | | | -0.017 | | | | | | | | 0.430 | | | | | | 0.248 | | | | | 0.255 | | | | | | -0.042 | | | | | 0.193 | | | | | | 0.183 | | | | | -0.137 | | | | | | | 0.128 | | | | | | 0.381 | | | | 0.282 | | | | | 0.155 | | | | 0.080 | | |
| Bengqiaodi | | | | | | | | | | | | | | | | | | | | | | | | | | | | | | | | | | | | | | | | | | | | | | | | | | | | | | | | | | | | | | | | | | | | | | | | | | | |
| Chemical composition (oxide wt%) | | | | | | | | | | | | | | | | | | | | | | | | | | | | | | | | | | | | | | | | | | | | | | | | | | | | | | | | | | | | | | | | | | | | | | | | | | | |
| SiO_2_ | | | | 0.40 | | | | 0.21 | | | 0.26 | | | | 0.37 | | | | 0.27 | | | 0.26 | | | | 0.27 | | | 0.26 | | | | 0.23 | | | | 0.25 | | | | 0.28 | | | 0.33 | | | | 0.28 | | | | 0.32 | | | 0.30 | | | |  |  |  |  |  |  |  |  |  |  |  |  |  |  |  |  |  |
| FeO | | | | 0.04 | | | | b.d.l | | | 0.02 | | | | 0.01 | | | | b.d.l | | | 0.05 | | | | 0.04 | | | 0.02 | | | | 0.07 | | | | 0.02 | | | | b.d.l | | | b.d.l | | | | 0.06 | | | | 0.00 | | | 0.01 | | | |  |  |  |  |  |  |  |  |  |  |  |  |  |  |  |  |  |
| CaO | | | | 53.35 | | | | 53.98 | | | 53.86 | | | | 54.19 | | | | 53.55 | | | 54.22 | | | | 54.00 | | | 54.65 | | | | 53.97 | | | | 53.78 | | | | 54.02 | | | 54.76 | | | | 54.41 | | | | 53.84 | | | 53.90 | | | |  |  |  |  |  |  |  |  |  |  |  |  |  |  |  |  |  |
| Na_2_O | | | | 0.03 | | | | 0.05 | | | 0.08 | | | | 0.04 | | | | 0.03 | | | 0.02 | | | | 0.09 | | | 0.05 | | | | 0.07 | | | | 0.06 | | | | 0.11 | | | 0.05 | | | | 0.06 | | | | 0.05 | | | 0.03 | | | |  |  |  |  |  |  |  |  |  |  |  |  |  |  |  |  |  |
| P_2_O_5_ | | | | 40.71 | | | | 41.50 | | | 41.98 | | | | 41.51 | | | | 41.97 | | | 42.28 | | | | 41.46 | | | 42.44 | | | | 41.06 | | | | 42.01 | | | | 42.00 | | | 41.60 | | | | 41.75 | | | | 41.75 | | | 41.39 | | | |  |  |  |  |  |  |  |  |  |  |  |  |  |  |  |  |  |
| MgO | | | | b.d.l | | | | b.d.l | | | b.d.l | | | | 0.01 | | | | 0.00 | | | b.d.l | | | | b.d.l | | | b.d.l | | | | 0.02 | | | | 0.04 | | | | 0.01 | | | b.d.l | | | | 0.01 | | | | 0.01 | | | b.d.l | | | |  |  |  |  |  |  |  |  |  |  |  |  |  |  |  |  |  |
| MnO | | | | 0.07 | | | | b.d.l | | | 0.07 | | | | 0.03 | | | | 0.09 | | | 0.09 | | | | 0.04 | | | 0.01 | | | | 0.13 | | | | 0.02 | | | | 0.12 | | | 0.01 | | | | 0.08 | | | | b.d.l | | | 0.09 | | | |  |  |  |  |  |  |  |  |  |  |  |  |  |  |  |  |  |
| SO_3_ | | | | 0.15 | | | | 0.15 | | | 0.14 | | | | 0.07 | | | | 0.06 | | | 0.21 | | | | 0.12 | | | 0.08 | | | | 0.24 | | | | 0.18 | | | | 0.19 | | | 0.28 | | | | 0.13 | | | | 0.11 | | | 0.20 | | | |  |  |  |  |  |  |  |  |  |  |  |  |  |  |  |  |  |
| F | | | | 2.86 | | | | 3.16 | | | 2.45 | | | | 1.96 | | | | 2.78 | | | 2.32 | | | | 3.26 | | | 2.65 | | | | 3.15 | | | | 2.73 | | | | 2.81 | | | 3.16 | | | | 2.25 | | | | 2.70 | | | 2.83 | | | |  |  |  |  |  |  |  |  |  |  |  |  |  |  |  |  |  |
| Cl | | | | 0.16 | | | | 0.20 | | | 0.21 | | | | 0.12 | | | | 0.18 | | | 0.18 | | | | 0.13 | | | 0.10 | | | | 0.20 | | | | 0.20 | | | | 0.15 | | | 0.15 | | | | 0.14 | | | | 0.13 | | | 0.23 | | | |  |  |  |  |  |  |  |  |  |  |  |  |  |  |  |  |  |
| Sub-Total | | | | 97.76 | | | | 99.25 | | | 99.07 | | | | 98.31 | | | | 98.93 | | | 99.63 | | | | 99.41 | | | 100.25 | | | | 99.15 | | | | 99.30 | | | | 99.68 | | | 100.33 | | | | 99.16 | | | | 98.91 | | | 98.99 | | | |  |  |  |  |  |  |  |  |  |  |  |  |  |  |  |  |  |
| O=F+Cl | | | | 1.24 | | | | 1.38 | | | 1.08 | | | | 0.85 | | | | 1.21 | | | 1.02 | | | | 1.40 | | | 1.14 | | | | 1.37 | | | | 1.20 | | | | 1.22 | | | 1.36 | | | | 0.98 | | | | 1.16 | | | 1.24 | | | |  |  |  |  |  |  |  |  |  |  |  |  |  |  |  |  |  |
| Total | | | | 96.52 | | | | 97.87 | | | 97.99 | | | | 97.45 | | | | 97.72 | | | 98.61 | | | | 98.00 | | | 99.11 | | | | 97.77 | | | | 98.10 | | | | 98.46 | | | 98.97 | | | | 98.19 | | | | 97.74 | | | 97.74 | | | |  |  |  |  |  |  |  |  |  |  |  |  |  |  |  |  |  |
| Si | | | | 0.034 | | | | 0.018 | | | 0.023 | | | | 0.031 | | | | 0.023 | | | 0.022 | | | | 0.023 | | | 0.022 | | | | 0.020 | | | | 0.021 | | | | 0.024 | | | 0.028 | | | | 0.024 | | | | 0.027 | | | 0.026 | | | |  |  |  |  |  |  |  |  |  |  |  |  |  |  |  |  |  |
| Fe | | | | 0.003 | | | |  | | | 0.002 | | | | 0.001 | | | |  | | | 0.003 | | | | 0.003 | | | 0.001 | | | | 0.005 | | | | 0.002 | | | |  | | |  | | | | 0.004 | | | | 0.000 | | | 0.000 | | | |  |  |  |  |  |  |  |  |  |  |  |  |  |  |  |  |  |
| Ca | | | | 4.955 | | | | 4.954 | | | 4.919 | | | | 4.955 | | | | 4.916 | | | 4.920 | | | | 4.948 | | | 4.936 | | | | 4.957 | | | | 4.915 | | | | 4.916 | | | 4.966 | | | | 4.947 | | | | 4.933 | | | 4.947 | | | |  |  |  |  |  |  |  |  |  |  |  |  |  |  |  |  |  |
| Na | | | | 0.006 | | | | 0.009 | | | 0.013 | | | | 0.006 | | | | 0.004 | | | 0.003 | | | | 0.014 | | | 0.008 | | | | 0.012 | | | | 0.010 | | | | 0.018 | | | 0.008 | | | | 0.010 | | | | 0.007 | | | 0.006 | | | |  |  |  |  |  |  |  |  |  |  |  |  |  |  |  |  |  |
| P | | | | 2.987 | | | | 3.010 | | | 3.030 | | | | 2.999 | | | | 3.045 | | | 3.032 | | | | 3.002 | | | 3.028 | | | | 2.980 | | | | 3.034 | | | | 3.020 | | | 2.980 | | | | 2.999 | | | | 3.023 | | | 3.002 | | | |  |  |  |  |  |  |  |  |  |  |  |  |  |  |  |  |  |
| Mg | | | |  | | | |  | | |  | | | | 0.001 | | | | 0.000 | | |  | | | |  | | |  | | | | 0.003 | | | | 0.004 | | | | 0.001 | | |  | | | | 0.002 | | | | 0.002 | | |  | | | |  |  |  |  |  |  |  |  |  |  |  |  |  |  |  |  |  |
| S | | | | 0.010 | | | | 0.010 | | | 0.009 | | | | 0.004 | | | | 0.004 | | | 0.013 | | | | 0.008 | | | 0.005 | | | | 0.016 | | | | 0.012 | | | | 0.012 | | | 0.018 | | | | 0.009 | | | | 0.007 | | | 0.013 | | | |  |  |  |  |  |  |  |  |  |  |  |  |  |  |  |  |  |
| Mn | | | | 0.005 | | | |  | | | 0.005 | | | | 0.002 | | | | 0.007 | | | 0.007 | | | | 0.003 | | | 0.000 | | | | 0.009 | | | | 0.001 | | | | 0.009 | | | 0.000 | | | | 0.006 | | | |  | | | 0.007 | | | |  |  |  |  |  |  |  |  |  |  |  |  |  |  |  |  |  |
| F | | | | 0.760 | | | | 0.839 | | | 0.650 | | | | 0.521 | | | | 0.737 | | | 0.615 | | | | 0.866 | | | 0.704 | | | | 0.836 | | | | 0.724 | | | | 0.746 | | | 0.838 | | | | 0.597 | | | | 0.716 | | | 0.751 | | | |  |  |  |  |  |  |  |  |  |  |  |  |  |  |  |  |  |
| Cl | | | | 0.023 | | | | 0.030 | | | 0.030 | | | | 0.018 | | | | 0.026 | | | 0.026 | | | | 0.019 | | | 0.014 | | | | 0.030 | | | | 0.030 | | | | 0.022 | | | 0.023 | | | | 0.020 | | | | 0.019 | | | 0.034 | | | |  |  |  |  |  |  |  |  |  |  |  |  |  |  |  |  |  |
| OH | | | | 0.216 | | | | 0.132 | | | 0.320 | | | | 0.461 | | | | 0.237 | | | 0.359 | | | | 0.115 | | | 0.282 | | | | 0.134 | | | | 0.246 | | | | 0.233 | | | 0.140 | | | | 0.383 | | | | 0.265 | | | 0.215 | | | |  |  |  |  |  |  |  |  |  |  |  |  |  |  |  |  |  |

All the Fe was taken as FeO. The formula calculation based on 8 cations. OH was calculated assuming stoichiometry, i.e. F+Cl+OH = 1 in the Z site. b.d.l means below the detection limit.

**Table 3** Trace elements of apatite (in ppm)

| Fuzishan | | | | | | | | | | | | | |
| --- | --- | --- | --- | --- | --- | --- | --- | --- | --- | --- | --- | --- | --- |
| Li | 6.14 | 2.50 | 2.93 | 1.15 | 2.00 | 2.05 | 2.32 | 3.88 | 2.07 | 0.72 | 4.10 | 2.09 | 6.33 |
| B | 2.20 | 2.45 | 2.47 | 2.25 | 2.38 | 2.44 | 2.47 | 2.50 | 2.52 | 2.68 | 2.52 | 2.43 | 2.54 |
| Sc | 0.13 | 0.14 | 0.21 | 0.28 | 0.01 | 0.16 | 0.20 | 0.34 | 0.02 | 0.18 | 0.25 | 0.31 | 0.32 |
| V | 28.1 | 26.7 | 22.3 | 15.0 | 16.2 | 14.6 | 21.7 | 28.1 | 32.9 | 13.1 | 25.9 | 23.7 | 40.7 |
| Cr | 0.12 | 0.95 | 0.40 | 0.00 | 0.45 | 0.00 | 0.00 | 0.80 | 0.80 | 4.63 | 0.70 | 0.18 | 0.64 |
| Mn | 559 | 558 | 497 | 463 | 464 | 584 | 456 | 477 | 473 | 506 | 583 | 543 | 482 |
| Co | 0.22 | 0.23 | 0.21 | 0.21 | 0.24 | 0.19 | 0.26 | 0.22 | 0.22 | 0.16 | 0.22 | 0.26 | 0.26 |
| Ni | 1.59 | 1.26 | 1.48 | 1.42 | 1.72 | 1.74 | 1.40 | 1.53 | 1.41 | 2.07 | 1.52 | 1.59 | 2.05 |
| Ga | 42.1 | 28.6 | 19.9 | 33.8 | 23.1 | 28.1 | 29.8 | 27.7 | 20.6 | 19.6 | 23.7 | 36.7 | 18.3 |
| Ge | 14.2 | 8.58 | 5.54 | 9.88 | 7.78 | 9.64 | 9.66 | 14.2 | 6.20 | 5.01 | 7.73 | 11.5 | 6.83 |
| Rb | 0.04 | 0.03 | 0.03 | 0.00 | 0.04 | 0.02 | 0.06 | 0.05 | 0.02 | 0.08 | 0.04 | 0.07 | 0.06 |
| Sr | 622 | 613 | 611 | 572 | 595 | 576 | 573 | 604 | 576 | 616 | 595 | 631 | 569 |
| Y | 155 | 101 | 80.0 | 126 | 114 | 129 | 135 | 203 | 74.5 | 79.5 | 105 | 177 | 103 |
| Zr | 1.28 | 1.06 | 0.60 | 1.16 | 1.41 | 0.78 | 0.82 | 0.68 | 0.70 | 0.81 | 0.85 | 1.53 | 1.00 |
| Ba | 0.30 | 0.41 | 0.39 | 0.20 | 0.32 | 0.31 | 0.33 | 0.35 | 0.18 | 0.37 | 0.74 | 0.27 | 0.18 |
| La | 2103 | 1786 | 1245 | 2105 | 1340 | 1684 | 1740 | 1429 | 1428 | 1455 | 1543 | 2354 | 1162 |
| Ce | 2737 | 2101 | 1401 | 2471 | 1573 | 2112 | 2323 | 2074 | 1618 | 1553 | 1924 | 2845 | 1406 |
| Pr | 209 | 150 | 102 | 178 | 117 | 161 | 181 | 201 | 114 | 104 | 146 | 213 | 109 |
| Nd | 657 | 456 | 314 | 550 | 380 | 521 | 596 | 841 | 342 | 299 | 451 | 657 | 348 |
| Sm | 75.3 | 46.5 | 35.5 | 57.0 | 44.8 | 57.9 | 66.9 | 129 | 35.4 | 30.5 | 49.3 | 77.0 | 43.9 |
| Eu | 18.3 | 14.4 | 10.8 | 19.3 | 11.3 | 12.1 | 14.0 | 11.6 | 14.5 | 11.6 | 11.5 | 18.8 | 8.87 |
| Gd | 62.9 | 41.1 | 30.3 | 47.7 | 39.0 | 47.3 | 52.1 | 107 | 29.5 | 27.7 | 40.1 | 64.0 | 38.4 |
| Tb | 5.78 | 3.71 | 2.73 | 4.45 | 3.97 | 4.38 | 4.78 | 10.3 | 2.75 | 2.43 | 3.86 | 6.13 | 3.70 |
| Dy | 25.8 | 16.7 | 13.3 | 20.6 | 18.6 | 21.2 | 22.7 | 44.4 | 12.3 | 12.0 | 17.2 | 30.4 | 17.7 |
| Ho | 4.79 | 3.20 | 2.44 | 3.84 | 3.51 | 3.95 | 4.28 | 7.41 | 2.34 | 2.23 | 3.32 | 5.48 | 3.17 |
| Er | 13.5 | 9.31 | 7.04 | 11.2 | 9.85 | 11.0 | 11.7 | 17.8 | 6.71 | 6.86 | 9.64 | 15.4 | 9.04 |
| Tm | 1.74 | 1.11 | 0.88 | 1.39 | 1.33 | 1.44 | 1.40 | 1.88 | 0.90 | 0.90 | 1.24 | 2.02 | 1.13 |
| Yb | 10.3 | 7.40 | 5.75 | 8.77 | 7.65 | 9.32 | 9.15 | 10.2 | 5.32 | 5.79 | 7.80 | 12.9 | 6.95 |
| Lu | 1.89 | 1.38 | 1.01 | 1.76 | 1.41 | 1.71 | 1.56 | 1.72 | 1.01 | 1.26 | 1.37 | 2.41 | 1.30 |
| Hf | 0.02 | 0.01 | 0.03 | 0.01 | 0.07 | 0.04 | 0.03 | 0.06 | 0.01 | 0.04 | 0.02 | 0.06 | 0.02 |
| Ta | 0.01 | 0.01 | 0.00 | 0.00 | 0.01 | 0.01 | 0.01 | 0.01 | 0.00 | 0.00 | 0.01 | 0.01 | 0.01 |
| W | 0.58 | 2.30 | 0.76 | 1.01 | 2.46 | 0.25 | 0.65 | 0.16 | 1.09 | 0.92 | 1.36 | 3.34 | 0.22 |
| Pb | 4.28 | 2.47 | 2.09 | 2.88 | 2.55 | 2.49 | 2.66 | 2.02 | 2.24 | 3.12 | 2.89 | 3.67 | 2.81 |
| Th | 90.9 | 88.1 | 59.2 | 147.8 | 97.1 | 133 | 89.7 | 65.6 | 85.3 | 87.9 | 60.3 | 152 | 71.6 |
| U | 30.0 | 23.1 | 29.0 | 80.9 | 49.6 | 56.7 | 38.9 | 21.9 | 34.7 | 51.9 | 25.2 | 86.7 | 26.2 |

| Niutoushan | | | | | | | | | | | | | | | |
| --- | --- | --- | --- | --- | --- | --- | --- | --- | --- | --- | --- | --- | --- | --- | --- |
| Li | 1.61 | 1.65 | 2.02 | 1.56 | 1.39 | 0.49 | 2.50 | 0.90 | 1.94 | 1.79 | 2.69 | 1.00 | 2.22 | 1.01 | 0.54 |
| B | 2.79 | 2.87 | 2.77 | 2.94 | 3.02 | 2.80 | 2.85 | 3.14 | 2.86 | 2.91 | 40.58 | 36.11 | 38.48 | 33.98 | 38.61 |
| Sc | 0.28 | 0.19 | 0.15 | 0.23 | 0.23 | 0.24 | 0.16 | 0.25 | 0.15 | 0.33 | 0.29 | 0.19 | 0.01 | 0.32 | 0.32 |
| V | 15.6 | 14.3 | 16.1 | 16.7 | 17.0 | 15.0 | 17.4 | 17.6 | 16.9 | 16.7 | 16.8 | 15.2 | 11.2 | 11.9 | 16.4 |
| Cr | 0.63 | 0.70 | 0.00 | 0.30 | 0.07 | 0.74 | 0.92 | 0.75 | 0.00 | 0.00 | 0.14 | 0.05 | 0.90 | 0.84 | 0.35 |
| Mn | 717 | 655 | 653 | 977 | 600 | 693 | 659 | 712 | 718 | 670 | 708 | 659 | 582 | 499 | 802 |
| Co | 0.52 | 0.48 | 0.51 | 0.52 | 0.50 | 0.55 | 0.49 | 0.40 | 0.46 | 0.44 | 0.47 | 0.47 | 0.41 | 0.45 | 0.49 |
| Ni | 1.92 | 1.80 | 1.92 | 2.07 | 2.08 | 2.13 | 2.02 | 1.81 | 1.71 | 1.85 | 1.89 | 1.80 | 2.04 | 1.97 | 1.96 |
| Ga | 56.6 | 40.3 | 41.5 | 46.0 | 48.9 | 41.2 | 34.5 | 32.0 | 40.8 | 39.6 | 49.6 | 41.6 | 19.5 | 48.4 | 46.1 |
| Ge | 19.2 | 15.3 | 14.0 | 14.6 | 16.1 | 12.9 | 13.8 | 9.64 | 13.2 | 12.3 | 49.3 | 43.4 | 17.8 | 56.0 | 45.5 |
| Rb | 0.03 | 0.02 | 0.03 | 0.08 | 0.03 | 0.01 | 0.03 | 0.09 | 0.04 | 0.04 | 0.04 | 0.05 | 0.01 | 0.06 | 0.02 |
| Sr | 457 | 433 | 466 | 444 | 439 | 459 | 445 | 463 | 475 | 482 | 454 | 454 | 512 | 418 | 455 |
| Y | 156 | 158 | 119 | 126 | 148 | 113 | 116 | 89.0 | 128 | 117 | 127 | 101 | 44.7 | 156 | 119 |
| Zr | 0.71 | 0.45 | 0.54 | 0.66 | 1.10 | 0.58 | 0.42 | 1.04 | 0.52 | 0.88 | 0.59 | 0.49 | 0.23 | 0.76 | 0.78 |
| Ba | 0.29 | 0.34 | 0.20 | 0.38 | 0.29 | 0.16 | 0.24 | 0.09 | 0.31 | 0.29 | 0.37 | 0.34 | 0.23 | 0.20 | 0.28 |
| La | 1688 | 1207 | 1298 | 1491 | 1634 | 1468 | 997 | 1229 | 1409 | 1439 | 1554 | 1401 | 646 | 1373 | 1504 |
| Ce | 2246 | 1524 | 1608 | 1844 | 1961 | 1672 | 1304 | 1363 | 1743 | 1694 | 1834 | 1582 | 697 | 1707 | 1718 |
| Pr | 175 | 124 | 124 | 135 | 146 | 120 | 107 | 97.8 | 131 | 124 | 134 | 112 | 49.8 | 136 | 122 |
| Nd | 564 | 446 | 402 | 427 | 457 | 374 | 389 | 311 | 418 | 390 | 452 | 369 | 164 | 504 | 404 |
| Sm | 65.8 | 63.9 | 48.5 | 49.8 | 55.4 | 42.3 | 53.1 | 35.2 | 49.6 | 44.8 | 54.7 | 42.7 | 18.5 | 66.1 | 48.3 |
| Eu | 12.5 | 10.3 | 10.9 | 13.2 | 14.1 | 10.3 | 7.65 | 8.93 | 10.9 | 12.6 | 13.4 | 12.2 | 5.17 | 11.5 | 11.9 |
| Gd | 62.8 | 61.0 | 43.9 | 46.8 | 51.6 | 41.1 | 50.8 | 33.4 | 45.7 | 41.3 | 50.9 | 38.7 | 16.5 | 59.4 | 43.6 |
| Tb | 5.84 | 6.24 | 4.27 | 4.47 | 5.03 | 3.78 | 4.91 | 3.06 | 4.34 | 3.80 | 4.72 | 3.58 | 1.51 | 6.09 | 4.16 |
| Dy | 26.9 | 30.5 | 20.1 | 20.9 | 25.4 | 18.0 | 22.5 | 14.7 | 20.8 | 18.8 | 22.2 | 17.2 | 7.14 | 28.1 | 20.1 |
| Ho | 5.24 | 5.56 | 3.75 | 4.09 | 4.76 | 3.47 | 4.07 | 2.80 | 4.13 | 3.67 | 4.20 | 3.48 | 1.41 | 5.45 | 3.92 |
| Er | 15.2 | 14.9 | 11.1 | 11.8 | 13.6 | 10.2 | 11.0 | 7.98 | 11.6 | 10.3 | 11.4 | 8.86 | 3.70 | 13.9 | 10.4 |
| Tm | 1.91 | 1.74 | 1.42 | 1.51 | 1.75 | 1.36 | 1.22 | 0.98 | 1.50 | 1.29 | 1.46 | 1.19 | 0.49 | 1.71 | 1.40 |
| Yb | 13.0 | 10.6 | 9.55 | 10.3 | 12.0 | 9.11 | 7.45 | 6.43 | 10.3 | 9.34 | 10.1 | 8.61 | 3.49 | 11.3 | 9.94 |
| Lu | 2.32 | 1.86 | 1.69 | 1.92 | 2.12 | 1.72 | 1.31 | 1.16 | 1.85 | 1.67 | 1.84 | 1.49 | 0.63 | 2.02 | 1.74 |
| Hf | 0.04 | 0.05 | 0.01 | 0.04 | 0.03 | 0.04 | 0.03 | 0.02 | 0.02 | 0.02 | 0.04 | 0.02 | 0.01 | 0.04 | 0.02 |
| Ta | 0.01 | 0.01 | 0.01 | 0.01 | 0.01 | 0.01 | 0.01 | 0.01 | 0.01 | 0.01 | 0.01 | 0.00 | 0.00 | 0.01 | 0.01 |
| W | 0.08 | 0.04 | 0.16 | 0.11 | 0.22 | 0.12 | 0.11 | 0.56 | 0.10 | 0.02 | 0.06 | 0.63 | 20.63 | 0.30 | 0.07 |
| Pb | 2.70 | 2.23 | 1.92 | 2.68 | 2.54 | 1.68 | 1.37 | 2.34 | 2.39 | 2.33 | 2.76 | 2.34 | 1.48 | 1.99 | 2.92 |
| Th | 57.4 | 35.9 | 47.7 | 54.8 | 86.4 | 47.2 | 33.4 | 91.9 | 42.5 | 78.2 | 46.5 | 40.4 | 35.2 | 57.1 | 64.3 |
| U | 8.8 | 7.1 | 11.9 | 10.3 | 20.5 | 9.4 | 6.0 | 23.9 | 7.1 | 20.4 | 8.4 | 8.7 | 10.5 | 17.1 | 14.5 |

| Ouyangshan | | | | | | | | | | | | | | | |
| --- | --- | --- | --- | --- | --- | --- | --- | --- | --- | --- | --- | --- | --- | --- | --- |
| Li | 5.12 | 2.98 | 5.11 | 4.08 | 4.41 | 4.88 | 2.33 | 7.16 | 7.99 | 4.55 | 4.73 | 4.90 | 6.67 | 6.05 | 6.25 |
| B | 25.65 | 24.20 | 26.96 | 26.25 | 26.66 | 26.55 | 28.00 | 27.22 | 28.19 | 27.30 | 29.15 | 30.13 | 28.88 | 28.52 | 28.99 |
| Sc | 0.25 | 0.38 | 0.16 | 0.15 | 0.17 | 0.25 | 0.25 | 0.30 | 0.16 | 0.15 | 0.17 | 0.22 | 0.14 | 0.21 | 0.24 |
| V | 42.5 | 36.3 | 40.2 | 36.4 | 37.9 | 34.4 | 36.5 | 35.8 | 47.3 | 37.9 | 43.4 | 33.9 | 40.8 | 37.4 | 44.3 |
| Cr | 1.30 | 1.51 | 0.88 | 0.47 | 0.00 | 1.89 | 0.55 | 2.02 | 0.00 | 0.00 | 0.00 | 0.00 | 0.00 | 0.24 | 0.00 |
| Mn | 397 | 468 | 467 | 513 | 489 | 431 | 473 | 519 | 429 | 478 | 428 | 507 | 466 | 519 | 454 |
| Co | 0.43 | 0.31 | 0.37 | 0.37 | 0.38 | 0.39 | 0.36 | 0.36 | 0.35 | 0.33 | 0.32 | 0.37 | 0.39 | 0.34 | 0.33 |
| Ni | 2.10 | 1.71 | 2.02 | 1.73 | 1.73 | 1.95 | 1.92 | 2.08 | 1.93 | 1.93 | 1.97 | 1.96 | 2.04 | 1.89 | 1.68 |
| Ga | 42.0 | 35.9 | 43.5 | 54.5 | 39.2 | 43.2 | 36.4 | 42.7 | 27.6 | 34.6 | 24.7 | 34.5 | 46.4 | 47.7 | 61.3 |
| Ge | 50.5 | 44.1 | 51.7 | 68.3 | 43.4 | 49.0 | 38.4 | 55.6 | 27.4 | 38.2 | 23.4 | 39.1 | 60.6 | 66.9 | 73.9 |
| Rb | 0.06 | 0.03 | 0.01 | 0.05 | 0.03 | 0.02 | 0.02 | 0.18 | 0.06 | 0.05 | 0.06 | 0.03 | 0.12 | 0.04 | 0.04 |
| Sr | 526 | 543 | 551 | 567 | 566 | 530 | 546 | 581 | 519 | 550 | 530 | 581 | 550 | 576 | 534 |
| Y | 153 | 111 | 135 | 202 | 113 | 124 | 89.8 | 164 | 72.8 | 107 | 58.1 | 108 | 169 | 194 | 231 |
| Zr | 0.45 | 0.51 | 0.36 | 0.54 | 0.36 | 0.59 | 0.58 | 0.43 | 0.44 | 0.52 | 0.53 | 0.37 | 0.54 | 0.33 | 0.75 |
| Ba | 0.19 | 0.12 | 0.15 | 0.17 | 0.10 | 0.09 | 0.05 | 0.49 | 0.05 | 0.16 | 0.13 | 0.17 | 0.13 | 0.13 | 0.09 |
| La | 1027 | 999 | 1124 | 1288 | 1092 | 1124 | 1072 | 1026 | 840 | 1016 | 820 | 919 | 1075 | 1009 | 1413 |
| Ce | 1520 | 1301 | 1585 | 1909 | 1432 | 1529 | 1355 | 1587 | 1075 | 1324 | 959 | 1276 | 1662 | 1690 | 2170 |
| Pr | 134 | 109 | 137 | 171 | 116 | 129 | 104 | 148 | 82.2 | 108 | 69.4 | 109 | 154 | 164 | 198 |
| Nd | 511 | 429 | 521 | 686 | 431 | 490 | 366 | 622 | 284 | 418 | 250 | 430 | 644 | 697 | 801 |
| Sm | 73.4 | 63.7 | 74.2 | 103 | 60.0 | 68.2 | 43.9 | 92.6 | 34.2 | 55.2 | 30.8 | 60.6 | 96.8 | 107 | 117 |
| Eu | 12.0 | 8.09 | 13.3 | 11.6 | 10.7 | 11.5 | 10.1 | 10.4 | 8.19 | 9.87 | 6.39 | 8.74 | 10.7 | 9.19 | 14.9 |
| Gd | 62.0 | 57.1 | 61.2 | 89.8 | 53.7 | 60.6 | 37.3 | 78.4 | 29.1 | 47.7 | 28.7 | 53.5 | 84.1 | 89.3 | 99.3 |
| Tb | 6.37 | 5.43 | 6.01 | 8.93 | 5.06 | 5.74 | 3.49 | 7.71 | 2.75 | 4.54 | 2.52 | 5.07 | 8.10 | 8.83 | 10.0 |
| Dy | 29.9 | 24.2 | 27.5 | 41.1 | 23.0 | 26.2 | 15.8 | 35.4 | 12.8 | 20.6 | 11.6 | 23.0 | 37.1 | 40.3 | 46.2 |
| Ho | 5.53 | 4.43 | 5.01 | 7.57 | 4.32 | 4.80 | 3.02 | 6.32 | 2.43 | 3.83 | 2.14 | 4.26 | 6.66 | 7.15 | 8.48 |
| Er | 13.6 | 10.3 | 12.0 | 18.2 | 10.4 | 11.4 | 7.77 | 14.9 | 6.41 | 9.41 | 5.24 | 10.3 | 15.9 | 16.8 | 20.8 |
| Tm | 1.72 | 1.13 | 1.39 | 2.15 | 1.21 | 1.35 | 0.99 | 1.66 | 0.84 | 1.12 | 0.60 | 1.19 | 1.85 | 1.87 | 2.42 |
| Yb | 11.4 | 7.07 | 8.69 | 13.6 | 7.73 | 8.53 | 6.77 | 10.2 | 5.77 | 7.39 | 3.94 | 7.32 | 11.0 | 10.6 | 15.7 |
| Lu | 1.78 | 1.17 | 1.39 | 2.06 | 1.29 | 1.37 | 1.22 | 1.52 | 0.97 | 1.30 | 0.72 | 1.18 | 1.65 | 1.61 | 2.47 |
| Hf | 0.05 | 0.03 | 0.02 | 0.06 | 0.03 | 0.03 | 0.03 | 0.06 | 0.03 | 0.04 | 0.02 | 0.03 | 0.04 | 0.07 | 0.05 |
| Ta | 0.01 | 0.01 | 0.00 | 0.01 | 0.01 | 0.00 | 0.00 | 0.01 | 0.00 | 0.01 | 0.00 | 0.01 | 0.01 | 0.02 | 0.01 |
| W | 0.10 | 0.21 | 0.24 | 0.20 | 0.10 | 0.19 | 0.23 | 0.06 | 0.15 | 0.18 | 0.69 | 0.21 | 0.11 | 0.09 | 0.12 |
| Pb | 2.44 | 2.49 | 2.39 | 3.71 | 2.54 | 2.46 | 2.62 | 4.77 | 2.24 | 2.74 | 2.46 | 2.83 | 2.78 | 2.27 | 2.93 |
| Th | 63.2 | 62.9 | 56.7 | 75.8 | 55.1 | 59.2 | 72.7 | 62.6 | 48.9 | 68.5 | 60.7 | 55.9 | 64.4 | 46.4 | 108 |
| U | 14.6 | 13.8 | 11.7 | 19.2 | 12.7 | 13.0 | 20.7 | 12.5 | 16.9 | 15.8 | 12.3 | 12.1 | 11.6 | 10.9 | 23.2 |

| Bengqiaodi | | | | | | | | | | | | | | | |
| --- | --- | --- | --- | --- | --- | --- | --- | --- | --- | --- | --- | --- | --- | --- | --- |
| Li | 0.30 | 0.85 | 0.45 | 0.28 | 0.40 | 0.48 | 0.55 | 0.29 | 0.27 | 0.24 | 0.44 | 0.46 | 0.17 | 0.89 | 0.45 |
| B | 2.04 | 2.28 | 2.23 | 2.08 | 2.04 | 2.14 | 2.35 | 2.22 | 2.49 | 2.16 | 2.42 | 2.31 | 1.58 | 2.59 | 2.50 |
| Sc | 0.55 | 0.51 | 1.03 | 0.55 | 0.65 | 0.68 | 0.66 | 0.59 | 0.41 | 0.57 | 0.63 | 0.65 | 0.69 | 0.68 | 0.70 |
| V | 11.4 | 13.5 | 12.0 | 12.8 | 15.5 | 13.5 | 10.8 | 15.5 | 11.2 | 10.9 | 14.0 | 14.2 | 11.1 | 11.8 | 14.1 |
| Cr | 0.00 | 0.00 | 0.10 | 0.13 | 0.10 | 0.01 | 0.00 | 0.32 | 0.00 | 0.00 | 0.40 | 0.13 | 1.22 | 0.27 | 0.00 |
| Mn | 656 | 753 | 750 | 647 | 853 | 868 | 866 | 734 | 704 | 803 | 603 | 636 | 616 | 626 | 721 |
| Co | 0.22 | 0.22 | 0.26 | 0.23 | 0.23 | 0.19 | 0.19 | 0.23 | 0.20 | 0.19 | 0.21 | 0.20 | 0.19 | 0.17 | 0.25 |
| Ni | 1.22 | 1.42 | 1.48 | 1.41 | 1.63 | 1.65 | 1.36 | 1.31 | 1.19 | 1.47 | 1.66 | 1.41 | 1.48 | 1.20 | 1.41 |
| Ga | 27.2 | 35.8 | 33.0 | 30.4 | 47.0 | 39.1 | 37.3 | 48.5 | 28.3 | 27.5 | 46.6 | 50.5 | 33.3 | 37.9 | 46.5 |
| Ge | 16.6 | 23.2 | 20.0 | 19.2 | 30.0 | 25.2 | 26.2 | 30.3 | 18.7 | 16.6 | 40.0 | 38.2 | 22.0 | 28.7 | 29.7 |
| Rb | 0.03 | 0.04 | 0.05 | 0.03 | 0.03 | 0.06 | 0.00 | 0.04 | 0.10 | 0.04 | 0.08 | 0.04 | 0.03 | 0.12 | 0.10 |
| Sr | 380 | 398 | 462 | 375 | 350 | 365 | 620 | 372 | 355 | 416 | 392 | 390 | 372 | 398 | 438 |
| Y | 197 | 257 | 226 | 242 | 328 | 273 | 274 | 357 | 229 | 200 | 855 | 606 | 282 | 449 | 320 |
| Zr | 0.46 | 1.06 | 0.67 | 0.66 | 1.51 | 1.13 | 0.86 | 1.21 | 0.53 | 0.69 | 0.86 | 0.98 | 0.43 | 0.56 | 1.26 |
| Ba | 0.19 | 0.30 | 0.33 | 0.21 | 0.15 | 0.30 | 2.03 | 0.23 | 0.27 | 0.42 | 0.12 | 0.17 | 0.41 | 0.19 | 0.37 |
| La | 1472 | 1824 | 1583 | 1556 | 2318 | 2198 | 1670 | 2485 | 1511 | 1545 | 1423 | 1858 | 1494 | 1442 | 2341 |
| Ce | 2058 | 2751 | 2515 | 2303 | 3531 | 3017 | 2751 | 3702 | 2137 | 2088 | 3129 | 3580 | 2443 | 2650 | 3589 |
| Pr | 197 | 268 | 244 | 226 | 344 | 282 | 293 | 356 | 205 | 198 | 405 | 415 | 250 | 308 | 353 |
| Nd | 691 | 923 | 807 | 796 | 1215 | 981 | 1070 | 1223 | 719 | 691 | 1647 | 1566 | 846 | 1168 | 1176 |
| Sm | 88.0 | 115 | 96.5 | 100 | 152 | 119 | 140 | 150 | 90.5 | 85.1 | 277 | 231 | 110 | 171 | 142 |
| Eu | 13.1 | 20.5 | 24.9 | 15.4 | 22.6 | 20.2 | 23.4 | 25.2 | 14.7 | 14.6 | 33.6 | 32.1 | 18.6 | 20.0 | 27.1 |
| Gd | 72.3 | 91.1 | 72.5 | 80.8 | 122 | 95.3 | 105 | 122 | 74.1 | 68.5 | 227 | 182 | 86.3 | 136 | 111 |
| Tb | 7.07 | 9.14 | 7.51 | 8.11 | 12.2 | 9.48 | 10.8 | 12.3 | 7.54 | 7.03 | 27.7 | 20.9 | 9.16 | 15.2 | 11.4 |
| Dy | 34.5 | 44.6 | 37.1 | 40.1 | 58.6 | 46.5 | 51.8 | 60.2 | 38.0 | 34.9 | 147 | 106 | 46.62 | 76.87 | 55.84 |
| Ho | 6.45 | 8.20 | 6.98 | 7.63 | 11.0 | 8.57 | 9.27 | 11.5 | 7.25 | 6.42 | 27.7 | 19.8 | 8.80 | 14.4 | 10.5 |
| Er | 17.0 | 22.4 | 19.2 | 20.9 | 29.2 | 23.6 | 23.6 | 32.0 | 20.0 | 17.1 | 76.3 | 53.5 | 24.4 | 39.4 | 27.8 |
| Tm | 2.10 | 2.84 | 2.67 | 2.59 | 3.63 | 2.96 | 3.04 | 4.13 | 2.55 | 2.11 | 10.42 | 7.05 | 3.23 | 5.12 | 3.64 |
| Yb | 13.0 | 18.3 | 18.0 | 17.1 | 22.6 | 18.6 | 17.6 | 26.3 | 16.3 | 14.0 | 65.5 | 47.2 | 21.3 | 33.9 | 23.1 |
| Lu | 2.16 | 2.86 | 2.83 | 2.83 | 3.64 | 3.13 | 2.73 | 4.70 | 2.83 | 2.34 | 9.93 | 7.29 | 3.55 | 5.36 | 3.66 |
| Hf | 0.04 | 0.04 | 0.02 | 0.04 | 0.03 | 0.05 | 0.07 | 0.06 | 0.04 | 0.02 | 0.14 | 0.10 | 0.04 | 0.05 | 0.05 |
| Ta | 0.01 | 0.01 | 0.01 | 0.01 | 0.01 | 0.01 | 0.01 | 0.02 | 0.00 | 0.01 | 0.03 | 0.03 | 0.01 | 0.01 | 0.01 |
| W | 0.10 | 0.06 | 0.06 | 0.14 | 0.13 | 0.09 | 0.06 | 0.23 | 0.13 | 0.08 | 0.15 | 0.19 | 0.13 | 0.17 | 0.14 |
| Pb | 1.30 | 1.72 | 1.71 | 1.27 | 2.15 | 2.15 | 1.87 | 2.14 | 1.46 | 4.04 | 1.60 | 1.57 | 7.43 | 1.56 | 1.92 |
| Th | 42.1 | 45.5 | 45.8 | 40.8 | 98.6 | 71.2 | 41.5 | 87.4 | 40.2 | 40.3 | 67.9 | 65.7 | 48.2 | 62.3 | 80.3 |
| U | 13.9 | 12.0 | 10.9 | 12.5 | 23.6 | 17.4 | 9.0 | 24.2 | 14.1 | 12.8 | 30.7 | 18.2 | 13.1 | 18.4 | 15.4 |

| Liujiawan | | | | | | | | | | | | | |
| --- | --- | --- | --- | --- | --- | --- | --- | --- | --- | --- | --- | --- | --- |
| Li | 0.37 | 0.23 | 0.23 | 0.31 | 0.15 | 0.28 | 0.21 | 0.18 | 0.19 | 0.53 | 0.39 | 0.34 | 0.19 |
| B | 1.94 | 2.18 | 2.50 | 2.38 | 2.26 | 2.11 | 2.41 | 2.74 | 2.12 | 2.16 | 1.95 | 2.10 | 2.13 |
| Sc | 0.59 | 0.35 | 0.58 | 0.34 | 0.48 | 0.47 | 0.44 | 0.74 | 0.51 | 0.54 | 0.56 | 0.36 | 0.56 |
| V | 11.7 | 12.3 | 10.3 | 11.5 | 13.7 | 10.5 | 12.9 | 15.4 | 12.1 | 11.1 | 14.8 | 11.6 | 12.2 |
| Cr | 0.00 | 0.00 | 0.16 | 0.40 | 0.54 | 0.13 | 0.00 | 0.17 | 0.00 | 0.32 | 0.00 | 0.12 | 0.36 |
| Mn | 684 | 490 | 576 | 701 | 353 | 677 | 406 | 585 | 558 | 996 | 1188 | 699 | 618 |
| Co | 0.29 | 0.25 | 0.22 | 0.19 | 0.28 | 0.25 | 0.24 | 0.20 | 0.24 | 0.32 | 0.35 | 0.22 | 0.25 |
| Ni | 1.33 | 1.48 | 1.31 | 1.63 | 1.16 | 1.38 | 1.64 | 1.45 | 1.58 | 1.09 | 1.51 | 1.63 | 1.16 |
| Ga | 24.3 | 21.3 | 22.4 | 26.1 | 20.6 | 26.6 | 21.9 | 44.5 | 23.0 | 27.7 | 49.4 | 23.5 | 25.4 |
| Ge | 15.0 | 11.8 | 13.3 | 15.9 | 11.4 | 15.7 | 11.6 | 29.6 | 12.4 | 15.6 | 28.6 | 13.3 | 15.0 |
| Rb | 0.00 | 0.00 | 0.00 | 0.08 | 0.01 | 0.10 | 0.00 | 0.16 | 0.01 | 0.01 | 0.02 | 0.01 | 0.02 |
| Sr | 529 | 577 | 338 | 397 | 474 | 649 | 392 | 344 | 475 | 884 | 1211 | 287 | 567 |
| Y | 152 | 127 | 147 | 180 | 135 | 162 | 136 | 426 | 147 | 186 | 300 | 154 | 163 |
| Zr | 0.76 | 0.55 | 0.45 | 0.60 | 0.60 | 0.62 | 0.65 | 1.93 | 0.54 | 1.07 | 1.37 | 0.54 | 0.66 |
| Ba | 1.23 | 0.65 | 0.24 | 0.32 | 0.21 | 1.27 | 0.12 | 0.25 | 0.52 | 1.08 | 3.49 | 0.28 | 0.79 |
| La | 1373 | 1352 | 1354 | 1399 | 1388 | 1473 | 1476 | 2348 | 1437 | 1448 | 2442 | 1453 | 1424 |
| Ce | 1850 | 1646 | 1713 | 1902 | 1605 | 1991 | 1713 | 3226 | 1759 | 2126 | 3737 | 1809 | 1930 |
| Pr | 175 | 144 | 160 | 188 | 138 | 189 | 146 | 333 | 158 | 201 | 361 | 163 | 181 |
| Nd | 590 | 478 | 547 | 659 | 456 | 633 | 476 | 1254 | 522 | 658 | 1197 | 551 | 614 |
| Sm | 71.5 | 57.6 | 66.3 | 84.5 | 54.9 | 73.9 | 55.7 | 187 | 62.9 | 78.1 | 141 | 67.3 | 75.8 |
| Eu | 13.1 | 11.6 | 9.73 | 12.9 | 9.77 | 14.0 | 10.9 | 27.6 | 11.8 | 17.2 | 29.9 | 10.6 | 13.1 |
| Gd | 57.4 | 46.4 | 53.3 | 66.5 | 47.0 | 59.3 | 47.3 | 158 | 50.7 | 60.3 | 109 | 54.8 | 60.6 |
| Tb | 5.57 | 4.53 | 5.19 | 6.56 | 4.54 | 5.94 | 4.51 | 16.8 | 4.94 | 6.23 | 10.9 | 5.43 | 5.94 |
| Dy | 26.4 | 21.1 | 24.5 | 31.6 | 22.6 | 27.8 | 21.3 | 80.8 | 23.4 | 29.2 | 51.7 | 25.7 | 27.7 |
| Ho | 4.86 | 3.94 | 4.66 | 5.80 | 4.28 | 5.12 | 4.16 | 15.0 | 4.74 | 5.77 | 9.68 | 5.00 | 5.34 |
| Er | 13.3 | 10.6 | 12.6 | 15.5 | 11.6 | 13.8 | 11.4 | 39.8 | 12.5 | 16.1 | 27.0 | 13.3 | 14.2 |
| Tm | 1.61 | 1.38 | 1.59 | 1.94 | 1.46 | 1.74 | 1.50 | 4.73 | 1.62 | 2.05 | 3.38 | 1.67 | 1.81 |
| Yb | 10.9 | 8.91 | 10.2 | 13.3 | 9.91 | 11.7 | 9.72 | 29.7 | 10.8 | 14.5 | 21.7 | 10.7 | 11.4 |
| Lu | 1.69 | 1.53 | 1.65 | 2.09 | 1.74 | 1.90 | 1.76 | 4.61 | 1.84 | 2.42 | 3.58 | 1.82 | 1.85 |
| Hf | 0.03 | 0.01 | 0.01 | 0.03 | 0.02 | 0.04 | 0.02 | 0.10 | 0.02 | 0.02 | 0.05 | 0.03 | 0.03 |
| Ta | 0.00 | 0.00 | 0.01 | 0.00 | 0.00 | 0.01 | 0.01 | 0.02 | 0.01 | 0.00 | 0.01 | 0.01 | 0.01 |
| W | 0.16 | 0.15 | 0.12 | 0.07 | 0.19 | 0.17 | 0.25 | 0.12 | 0.05 | 0.22 | 0.13 | 0.12 | 0.66 |
| Pb | 1.15 | 1.16 | 1.09 | 1.22 | 1.05 | 1.42 | 1.10 | 2.08 | 1.07 | 2.63 | 3.73 | 1.19 | 1.23 |
| Th | 35.7 | 33.3 | 30.1 | 40.9 | 41.1 | 36.3 | 40.8 | 108 | 36.9 | 43.6 | 85.7 | 36.1 | 37.7 |
| U | 9.6 | 8.4 | 9.3 | 11.1 | 10.3 | 8.7 | 14.2 | 26.7 | 9.4 | 8.5 | 13.6 | 12.2 | 8.7 |
